# Mimicking Nonribosomal Peptides from the Marine Actinomycete *Streptomyces* sp. H-KF8 Leads to Antimicrobial Peptides

**DOI:** 10.1021/acsinfecdis.3c00206

**Published:** 2023-12-19

**Authors:** Luisa I. Beyer, Ann-Britt Schäfer, Agustina Undabarrena, Inger Mattsby-Baltzer, Daniel Tietze, Elin Svensson, Alexandra Stubelius, Michaela Wenzel, Beatriz Cámara, Alesia A. Tietze

**Affiliations:** † Department of Chemistry and Molecular Biology, Wallenberg Centre for Molecular and Translational Medicine, University of Gothenburg, Medicinaregatan 7B, Gothenburg 413 90, Sweden; ‡ Department of Life Sciences, Chalmers University of Technology, Kemigården 4, Göteborg 412 96, Sweden; § Center for Antibiotic Resistance Research in Gothenburg, University of Gothenburg, Box 100, Göteborg 405 30, Sweden; ∥ Departamento de Química & Centro de Biotecnología Daniel Alkalay Lowitt, Laboratorio de Microbiología Molecular y Biotecnología Ambiental, Universidad Técnica Federico Santa María, Valparaíso 2340000, Chile; ⊥ Department of Infectious Diseases, Institute of Biomedicine, The Sahlgrenska Academy at University of Gothenburg, University of Gothenburg, Box 440, Göteborg 405 30, Sweden

**Keywords:** antimicrobial peptides, *Streptomyces*, nonribosomal peptides, cyclic peptides, secondary metabolite, marine sediments

## Abstract

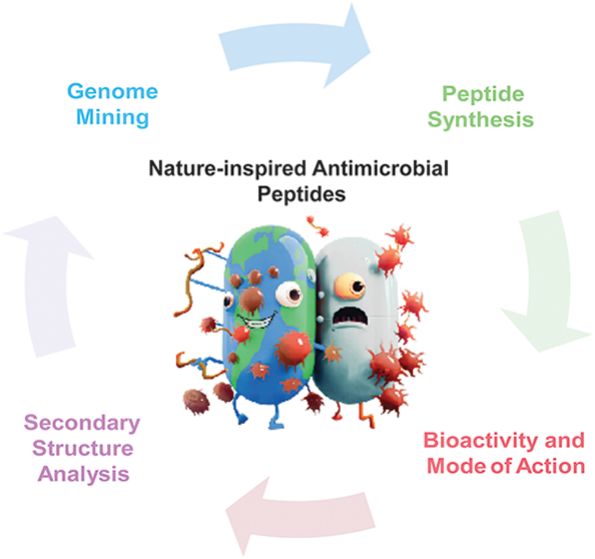

Microorganisms within the marine environment have been shown to be very effective sources of naturally produced antimicrobial peptides (AMPs). Several nonribosomal peptides were identified based on genome mining predictions of *Streptomyces* sp. H-KF8, a marine Actinomycetota isolated from a remote Northern Chilean Patagonian fjord. Based on these predictions, a series of eight peptides, including cyclic peptides, were designed and chemically synthesized. Six of these peptides showed antimicrobial activity. Mode of action studies suggest that two of these peptides potentially act on the cell membrane via a novel mechanism allowing the passage of small ions, resulting in the dissipation of the membrane potential. This study shows that though structurally similar peptides, determined by NMR spectroscopy, the incorporation of small sequence mutations results in a dramatic influence on their bioactivity including mode of action. The qualified hit sequence can serve as a basis for more potent AMPs in future studies.

Antimicrobial resistance is one of the most serious public health threats nowadays, and combating pathogenic resistant bacteria is urgently needed.^1^ Antimicrobial peptides (AMPs) represent a novel class of antimicrobial agents^2^ that are produced by living organisms as nonspecific innate immune system modulators.^3^ AMPs usually represent the first-line defense system showing direct microbicidal effects against many bacteria, fungi, parasites, and/or viruses.^4^ They show a broad variety of structures and modes of action. Since peptides are metabolized to amino acids, they are biodegradable, and are known to exhibit slower resistance development rates compared to commercial small-molecule antibiotics due to their more complex modes of action.^5^ The microbicidal mechanisms of AMPs vary considerably, comprising nonspecific cell membrane disruption, specific binding to membrane- or cell wall-bound targets, interaction with intracellular targets, or even with multiple targets.^6^ The nonspecific membrane interactions are the most commonly described in the literature, though these AMPs usually do not end up as promising drug candidates.^3^ In contrast, AMPs with more specific targets, either located on the cell surface or inside the cells, are of immense interest for drug development. Therefore, not only the discovery of structurally new compounds but also studying their mode of action is a crucial part in the development of AMPs as potential new drug candidates.

The number of natural AMPs is expected to be in the range of several millions,^7^ but to date only 18 000 validated AMPs are reported in public databases, of which even fewer reached clinical trials so far.^8^ One possible approach to boost the bioprospection of novel AMPs is the exploration of understudied environments, like the marine niche (Figure 1). Indeed, several microorganisms within the marine environment have demonstrated to be very effective sources of naturally produced AMPs.^9^ Their metabolic strategies are adapted to extreme conditions with large temporal and spatial variability.^10^ Samplings along the vast Chilean coastline for members of the Actinobacteria phylum have been reported to produce novel bioactive metabolites.^11−13^ The Comau Fjord, located in Northern Chilean Patagonia, is a suitable environment to explore the diversity and antimicrobial potential of unique marine bacteria, especially those belonging to the Actinomycetota phylum, like *Streptomyces*, a well-known antibiotic-producer genus. *Streptomyces* sp. HKF8 harbors a promising metabolic repertoire due to its phenotypic adaptations. To name a few, it was isolated from 15 m-deep marine sediments, requiring seawater for growth, and tolerates high salt concentrations and low temperatures.^12^

**Figure 1:**
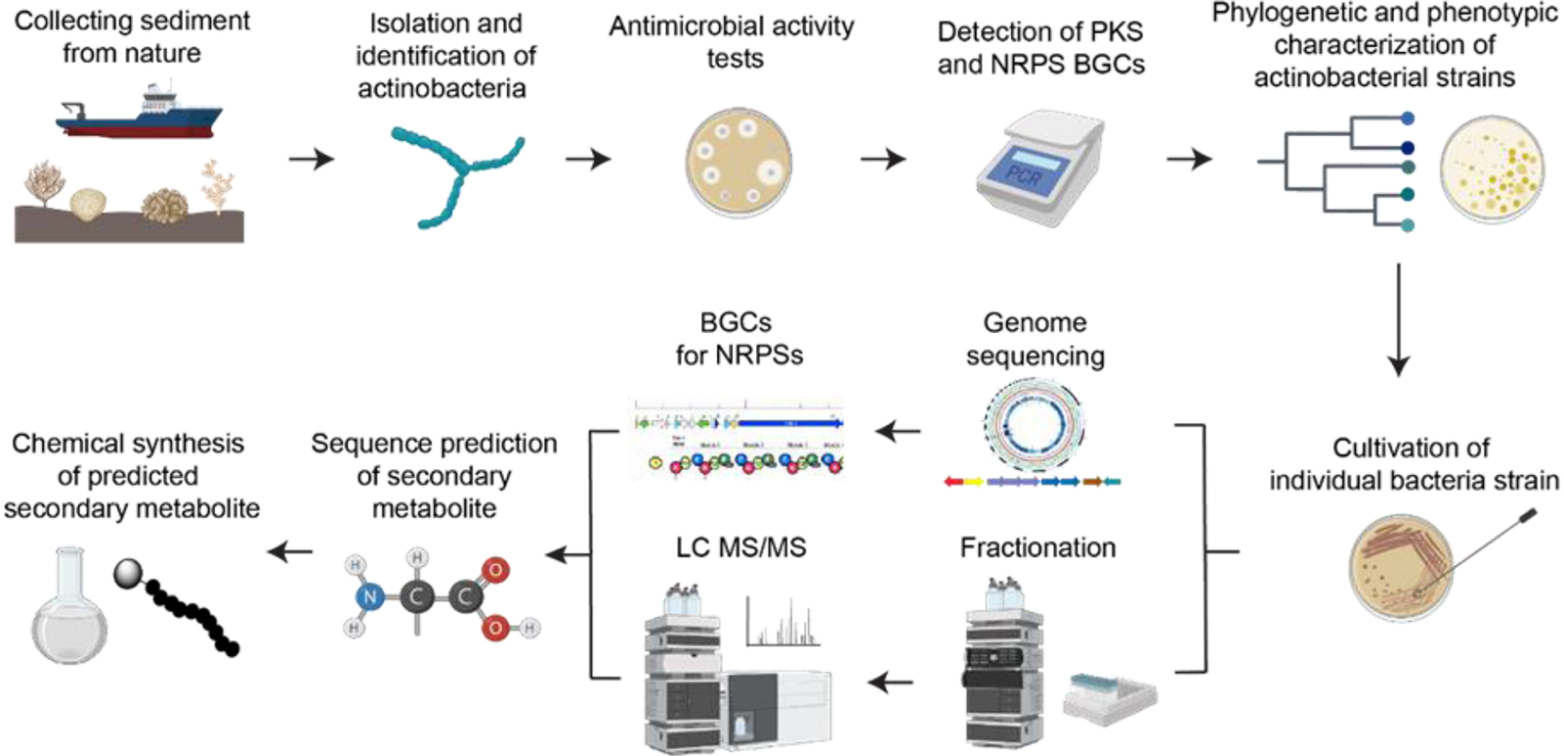
Marine natural nonribosomal antimicrobial peptide discovery pipeline.

In previous reports, the genome sequencing of the strain *Streptomyces* sp. H-KF8 led to the assembly of 11 scaffolds, representing a 7.6 Mbp linear chromosome.^14^ With the help of the antiSMASH v3.0^15^ 26 biosynthetic gene clusters (BGCs) for specialized metabolites were identified, among which 81% represent low similarities to already known BGCs registered in the Minimum Information about a Biosynthetic Gene cluster (MIBiG^16^) repository.^16^ Remarkably, the two nonribosomal peptide synthetases (NRPSs) detected in *Streptomyces* sp. HKF8’s genome showed a very low similarity to known pathways (mannopeptimycin, 7% similarity to BGC0000388, and streptolydigin, 13% similarity to BGC0001046).^17^ These studies uncovered the genetic potential of this strain to produce novel antimicrobial compounds, through NRPS pathways.^12^

The NRPSs are responsible for the synthesis of peptides composed of proteinogenic and nonproteinogenic amino acids that can either present a linear or cyclic structure.^18^ The latter is of special interest due to the possibility of overcoming structural and protease instability issues.^19^ Additionally, NRPS pathways exhibit very complex chemistry in terms of the diversity of their functional groups.^20,21^ Following the discovery pipeline (Figure 1), the prediction of the core skeleton of novel peptides followed by their chemical synthesis, bioactivity, and structural analysis, as well as mode of action belong to the key steps of characterizing nonribosomal AMPs.

In this study, we report the discovery, design, and development of novel naturally inspired AMPs. A series of linear and cyclic antimicrobial peptides, based on genomic data predictions of the marine Actinomycetota *Streptomyces* sp. H-KF8, were synthesized and characterized with a focus on determination of the influence of amino acid composition on their secondary structure and mode of action, leading to activity against both Gram-positive and Gram-negative bacteria, as well as yeast. Through this journey, bioactive qualified hit structures have been identified among a set of structurally similar peptides, which can lead to potent AMPs in future studies.

## Results and Discussion

### Genome Mining of *Streptomyces* sp. H-KF8

**Figure 2:**
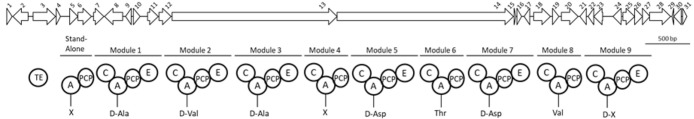
Genetic representation of NRPS #1.8 BGC of *Streptomyces* sp. H-KF8. Genes composing the BGC are numbered, and their predicted function can be found in Table S1. The biosynthetic modules of the two NRPS genes (numbered as 13 and 14) are shown in detail: A, adenylation domain; C, condensation domain; PCP, peptidyl carrier protein; E, epimerization domain; and TE, thioesterase domain. The amino acid that is predicted to be incorporated in the assembly line by every A-domain is shown as follows: Ala, alanine; Val, valine; Asp, aspartic acid; Thr, threonine; and X, unknown. Bar is on a scale corresponding to gene size.

An updated genome mining analysis was performed using antiSMASH v6.0.^22^ and confirmed the low similarity of both NRPS biosynthetic gene clusters mentioned above.^17^ Among them, the NRPS BGC #1.8 presented novel genetic features, and therefore, was selected for further analysis (Figure 2 and Table S1).

The NRPS #1.8 (Figure 2) is composed of 31 genes arranged in a BGC of 77 237 bp of total length. It harbors two *nrps* biosynthetic genes, where 10 adenylation domains (A-domain) were detected, nine representing complete modules and one stand-alone domain. A thioesterase domain (TE-domain) was found contiguous to the *nrps* genes, suggesting a final step of releasing and cyclization of the peptide chain. Genomic prediction suggested that the putative product of this pathway could be a decapeptide with six d-amino acids, due to the presence of six epimerization domains (E-domain) within *nrps* genes of this BGC (Figure 2). Moreover, the analysis indicates that this BGC has more than one resistance protein. This could indicate that the peptide formed has more than one possible mechanism for inhibition. To confirm the bioinformatic prediction and identify the peptide’s primary structure, LC-MS analysis of the bioactive extract of *Streptomyces* sp. H-KF8 was conducted (Figure S1). MALDI-TOF MS/MS confirmed the presence of 8 out of the 10 predicted amino acids, leading to the prediction of the following consensus sequence (Figure S2).

X_1_ – _d_Ala – _d_Val – _d_Ala–Trp – _d_Orn – X_7_ – _d_Orn–Val – _d_Tyr

This consensus sequence presents variability in some positions of the assembly line. For instance, the presence of a stand-alone A-domain could indicate that X_1_ may have a nonamino acidic nature, which is consistent with the absence of its detection by MALDI-TOF MS/MS. Additionally, antiSMASH was not able to predict the two noncanonical amino acids that are being incorporated in the assembly line (i.e., d-Orn), although they were detected by MALDI-TOF MS/MS and successfully predicted by the complementary bioinformatic tool PRISM.^23^ Moreover, position X_10_ could be a d-Tyr or a tyrosine modified with a nitro group (NO_2_-Tyr), due to the presence of tailoring enzymes within the BGC responsible for this modification. Finally, the genetic predictions related to the NRPS #1.8 and the functional evidence of the formation of a peptide in crude extracts of strain H-KF8 suggests that the predicted peptide core could be further decorated with sugar or amino-sugar moieties, indicating that *Streptomyces* sp. H-KF8 is able to produce a natural product of complex nature, putatively a cyclic glycodecapeptide. Further chemical diversity based on the presence of noncanonical amino acids and epimerization domains is conceivable. All of the above-mentioned hypotheses will remain to be confirmed; however, we used this consensus sequence as a starting point to synthesize naturally inspired bioactive peptides that could be proposed as novel therapeutic agents.

### Peptide Design and Synthesis

**Figure 3:**
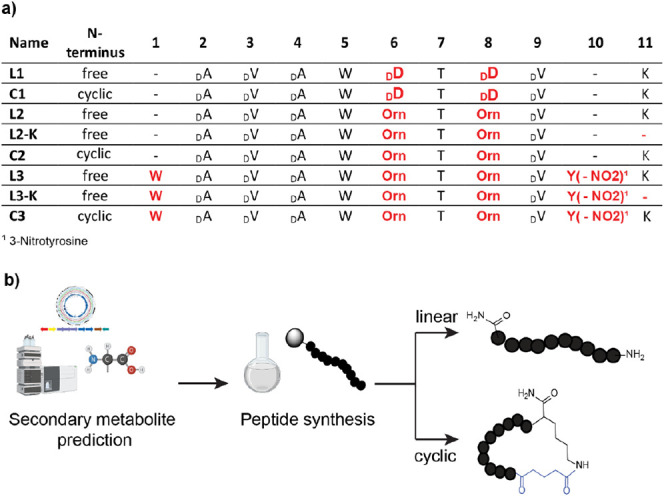
Design of peptides. (a) Sequences of synthesized nonribosomal peptides. (b) Schematic representation of identification and synthesis strategy workflow of NRP secondary metabolites.

Based on the predicted consensus sequence, five different linear peptides were generated (**L1, L2, L2-K, L3**, and **L3-K**, Figure 3a). The presence of the TE-domain suggested the presence of cyclic peptides. Therefore, three cyclic versions were synthesized, representing the cyclic forms of peptides **L1, L2**, and **L3** (**C1–C3**, Figure.3b).

Since, no certain prediction could be done for the *C*- and *N*-terminal amino acids, the first peptide contained only eight amino acids (**L1**, Figure 3a) with respect to the predicted core sequence. For the second peptide d-Asp was exchanged with ornithine (Orn) (**L2**, Figure 3a), due to the possible variation in the PRISM analysis. The third peptide was composed of 10 amino acids, where the first amino acid is Trp, and the tenth amino acid is Tyr-NO_2_ (**L3**, Figure 3a). For the on-resin head-to-tail cyclization via side chain (Figure 3b), a *C*-terminal Lys was introduced, while glutaric anhydride was coupled to the *N*-terminus, which results in a carboxy group.^24^

For comparison regarding the cyclic peptides, two linear peptides **L2-K** and **L3-K** were synthesized, which carried no C-terminal Lys, aiming to study the influence of positively charged amino acid at the C-terminus.

All designed peptides were synthesized following the standard Fmoc-based solid-phase peptide synthesis (SPPS) protocol, purified, and characterized by RP-HPLC, LC-MS, and amino acid analysis (Figures S3–S11, Tables S2,S3). Analytical data and physicochemical properties of the synthesized peptides are summarized in Tables 1 and S2. Amino acid analysis revealed that the peptide content was around 94%, which was considered for concentration calculations.

**Table 1:** Physicochemical Properties of Synthesized Peptides

	physicochemical properties					
peptide	AA number	net charge^a^	*m*/*z* calc. [M+2H]^+^^b^	*m*/*z* measured [M+2H]^+^^b^	*R*_t_^b^ (min)	RMSD (Å)
L1	9	0	502.26	502.26	2.6	1.2
C1	9	0	550.27	550.27	4.0	n.d.
L2	9	+4	501.31	501.31	1.9	1.5
L2-K	8	+3	437.27	437.27	2.0	n.d.
C2	9	+2	549.32	549.32	3.2	0.6
L3	11	+4	698.38	698.38	4.8	0.8
L3-K	10	+3	634.33	634.33	5.0	n.d.
C3	11	+2	746.39	746.39	5.9	1.9

a Net charge is calculated at pH 7 from theoretical p*K*_a_ values.

b Values were obtained from RP-HPLC, gradient 10–50% of eluent B (acetonitrile containing 0.1% TFA) in 10 min at a flow rate of 2 mL/min.

### Antimicrobial Activity

Bioactivity of chemically synthesized AMPs is usually determined by applying antimicrobial susceptibility testing (e.g., broth dilution testing) to determine the minimum inhibitory concentration (MIC), which is standardized for small molecules.^25^ However, in AMP discovery, this approach faces limitations since many peptides by nature are not as stable as small organic molecules and the complex media composition suitable for bacterial growth in the lab may (i) affect a peptide’s bioactivity and (ii) not represent the actual infection environment.^26,27^ As a consequence, many potent AMPs can be mistakenly discarded and compounds with a novel mode of action and novel targets will be overlooked, which is detrimental in the face of the current antimicrobial resistance crisis. Therefore, the research community is adapting the conditions to determine the bioactivity of AMPs, which can make it difficult to compare data in the literature landscape from one discovery to another and from small molecules to peptides.^26^

One of the alternative and reliable methods is to determine AMPs’ minimal microbicidal concentration, i.e., the lowest concentration killing 99% of the inoculum (MMC_99_),^28^ which is presented in this study alongside the MIC values. The antimicrobial activities of the peptides were investigated against Gram-positive *S. aureus*, Gram-negative *E. coli*, and the yeast *Candida albicans*. **L2**, **L3**, **L3-K**, and **C3** showed antimicrobial activity against the tested microorganisms (Table 2). The values obtained for new peptides were compared with well-known antimicrobials, such as fusidic acid, polymyxin B, and clotrimazole.

**Table 2:** Antimicrobial Activity^a^

	*S. aureus*				*E. coli*				*C. albicans*			
	MMC_99_ (μg/mL)				MMC_99_ (μg/mL)				MMC_99_ (μg/mL)			
peptide	2 h	6 h	24 h	MIC (μg/mL)	2 h	6h	24 h	MIC (μg/mL)	2 h	6 h	24 h	MIC (μg/mL)
L1	>100	>100	>100	>512	>100	>100	>100	>512	>100	>100	>100	>512
C1	>100	>100	>100	>512	>100	>100	>100	>512	>100	>100	>100	>512
L2	(100)^b^	(100)^b^	>100	>512	>100	>100	>100	>512	50	25	25	>512
L2-K	>100	>100	>100	>512	>100	>100	>100	>512	>100	>100	>100	>512
C2	>100	>100	>100	>512	>100	>100	>100	>512	>100	>100	>100	>512
L3	12.5	12.5	25	248	12.5	6.3	6.3	64	12.5	6.3	12.5	70.3
L3-K	25	25	25	418.7	50	25	25	136.6	25	25	12.5	248
C3	>100	>100	>100	>512	>100	>100	100	512	>100	>100	>100	>512
fusidic acid	25	6.3	1.6	0.09								
PolB^c^					<1.6	<1.6	<1.6	2.3				
clotrimazole									>100^d^	>100^d^	>100^d^	4

a Median values, *n* = 3. >100, killing of 99% of the inoculum was not achieved with the highest concentration of the peptide (100 μg/mL). >512, inhibition of visible growth (MIC) was not achieved with the highest concentration of peptide (512 μg/mL).

b (100), just below 99% killing (98.5%) at 100 μg/mL.

c PolB: polymyxin B.

d Colonies grew slowly at all three time points.

The MMC_99_ study (Table 2) shows that while **L2** was active against *C. albicans* at an MMC_99_ of 25 μg/mL and much less active against *S. aureus*, **L3** and **L3-K** were found to be active against all three species. **L3** was the most active peptide against *S. aureus*, *E. coli*, and *C. albicans*, showing MMC_99_ values ranging from 6.3 to 25 μg/mL. **L3-K**, lacking the C-terminal lysine, which was introduced for the cyclization of **L3**, still showed activity, although at higher concentrations of 12.5–50 μg/mL. Interestingly, upon cyclization (**C3**) the peptide lost again activity, now being only active against *E. coli* at concentrations ≥ 100 μg/mL. It can be concluded that the length and charge of the linear peptides (Table 1) have only a minor impact on antimicrobial activity, while structural changes, caused by the sequence variations and cyclization, are most likely the reasons behind altered antibacterial activity. Taken together, the peptides **L3** and **L3-K** show the most promising minimum microbicidal concentrations for all three strains tested, compared to the well-known commercially available antimicrobials. Fusidic acid, with its time decreasing MMC_99_ value for *S. aureus* from 25 to 1.6 μg/mL, shows different kinetics over time compared to new compounds **L3** and **L3-K**, where the MMC_99_ value stays constant over 24 h or slightly increases, pointing to the differences in mechanism of action. Polymyxin B outperforms by its MMC_99_ values in *E. coli*, though low MMC_99_ values for peptides **L3** and **L3-K** and its decreased values over time suggest here as well the differences in mechanism of action. In contrast, clotrimazole shows considerably higher MMC_99_ values compared to **L3**, **L3-K**, and **L2**, indicating that new peptides possess different mode of action against *C. albicans*.

The MIC values for all organisms studied are expectedly noticeably higher than the MMC_99_ values due to the media used. The lowest MIC values were observed for **L3** peptide at concentration of 64 μg/mL in *E. coli*, 70 μg/mL in *C. albicans*, and 248 μg/mL in *S. aureus*. The values are raising higher when the Lys is absent in the peptide sequence (Table 2), which represents similar trend observed for the MMC_99_ values. MIC values were considered for further experiments as mode of action studies, hemolysis and cytotoxicity of studied peptides.

### Salt Resistance of Peptides

Salt sensitivity is one of the well-known limiting factors that influence microbicidal activity of AMPs and limits their initial application as novel antibiotics,^29^ a problem that can be circumvented by using a peptidomimetic approach.^5^ Here, the newly identified natural peptides were studied to determine their initial salt stability. Biological salt stability was tested for the two most potent peptides, **L2** and **L3**, by adding either 85 or 150 mM NaCl to the growth medium of the MMC_99_ assay (Table S6). While **L2** completely lost its activity in the presence of both salt concentrations, **L3** retained moderate activity (MMC_99_ 50–200 μg/mL) at 85 mM NaCl against *C. albicans*, but not against *E. coli*, or *S. aureus*. These results show that the peptides are not salt resistant, which seems to be surprising because these peptides were predicted from the seawater organism *Streptomyces* sp. HKF8. However, this might be a consequence of simplifying the predicted structures to the peptide core or the uncertainty of the structure predictions based on the genome analysis.

### Peptide Stability in Serum

Peptide stability in serum is another limiting factor for AMPs as novel antibiotics.^30^ The peptide stability of the synthesized peptides (**L2**, **L3**, **L3-K**, and **C3**) was investigated in human serum using HPLC after 0, 0.5, 1, 4, and 24 h. The results show that the peptides are stable after 24 h of incubation in human serum at 37 °C, indicated by the consistent signal of the individual peptide in their chromatograms (Figure S13). After 24 h, the chromatograms for **L2**, **L3**, and **L3-K** show slight peak shape differences. **L2** develops a small shoulder with an overall volume percentage of 0.4%. The chromatograms of **L3** and **L3-K** show small additional peaks with a total volume of 0.6% (**L3**) and 1.3% (**L3-K**). The chromatogram for **C3** does not show any additional signal after 24 h.

### Hemolysis

Since antimicrobial peptides are known to disturb the cell membrane integrity, their hemolytic activity on human erythrocytes has been used as an indication of their toxicity. The hemolytic activity of the synthesized peptides **L2**, **L3**, **L3-K**, and **C3** was tested against fresh human erythrocytes from blood donors post peptide exposure (Figure S14). The hemolytic activity of the four peptides was performed in PBS buffer at pH 5, since **L3**, **L3-K**, and **C3** developed a clear yellow color at pH 7.4 in PBS buffer, due to an internal hydrogen bond formation related to Y-NO_2_ with a p*K*_a_ value of 7.1.^31^ The lower pH value removed the yellow color while leaving the hemoglobin absorbance unaffected. As a result of the study, only **L3** exceeded the background level with a hemolytic activity of 4% at the highest concentration (Figure S14). Additionally, the absence or very low hemolytic activity is to be expected since all the peptides were sensitive to physiological salt concentration.

### Cytotoxicity

**Figure 4:**
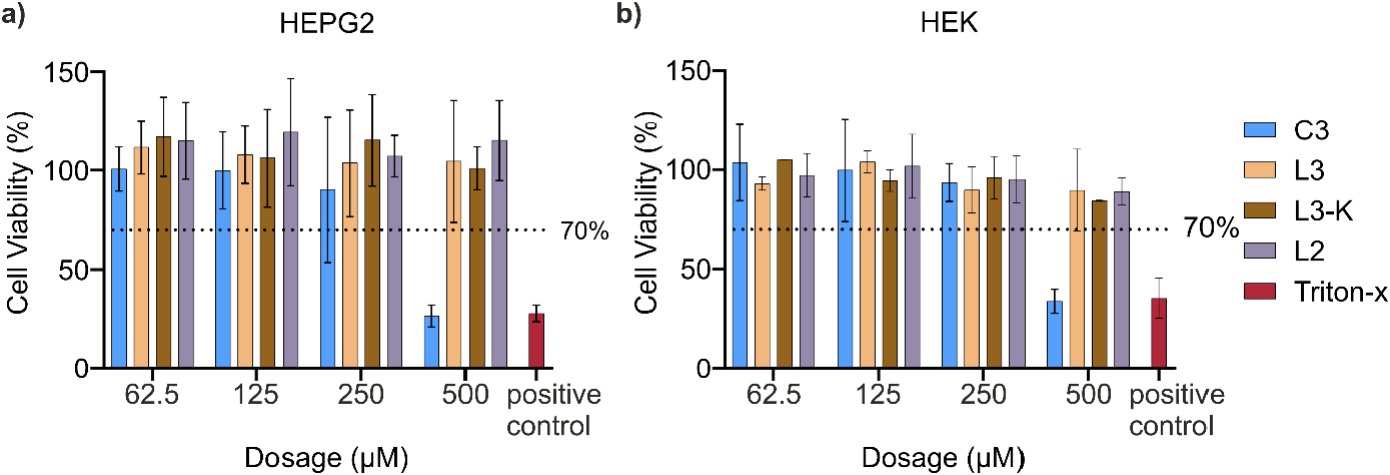
Cell viability assessment using the resazurin assay 24 h post peptide exposure: (a) HepG2 and (b) HEK cells were treated with various concentrations of the peptides **C3**, **L3**, **L3-K**, and **L2**. Triton-X was used as a positive control. Cells cultured in pure cell media were used as negative controls and were considered to have 100% cell viability. The line represents the threshold for cytotoxicity level of 70% of cell viability compared to the negative control. Data are presented as the mean ± SD of three biological replicates of the HepG2 cells and the mean ± SD of two biological replicates of the HEK cells.

To confirm the peptide selectivity toward bacteria cells, cytotoxicity assays of synthesized peptides **L2**, **L3**, **L3-K**, and **C3** against human embryonic kidney (HEK) and hepatoblastoma (HepG2) were performed (Figure 4). The cell viability was assessed using resazurin 24 h post peptide exposure. The line at 70% cell viability marks out the threshold for cytotoxic potential compared to the negative control. Peptides **L2**, **L3**, and **L3-K** showed cell viability significantly greater than the threshold, indicating no cytotoxicity at any tested concentration. Only **C3** at the highest tested concentration seems to have cytotoxic properties with cell viability similar to the positive control.

### Secondary Structure Elucidation

**Figure 5:**
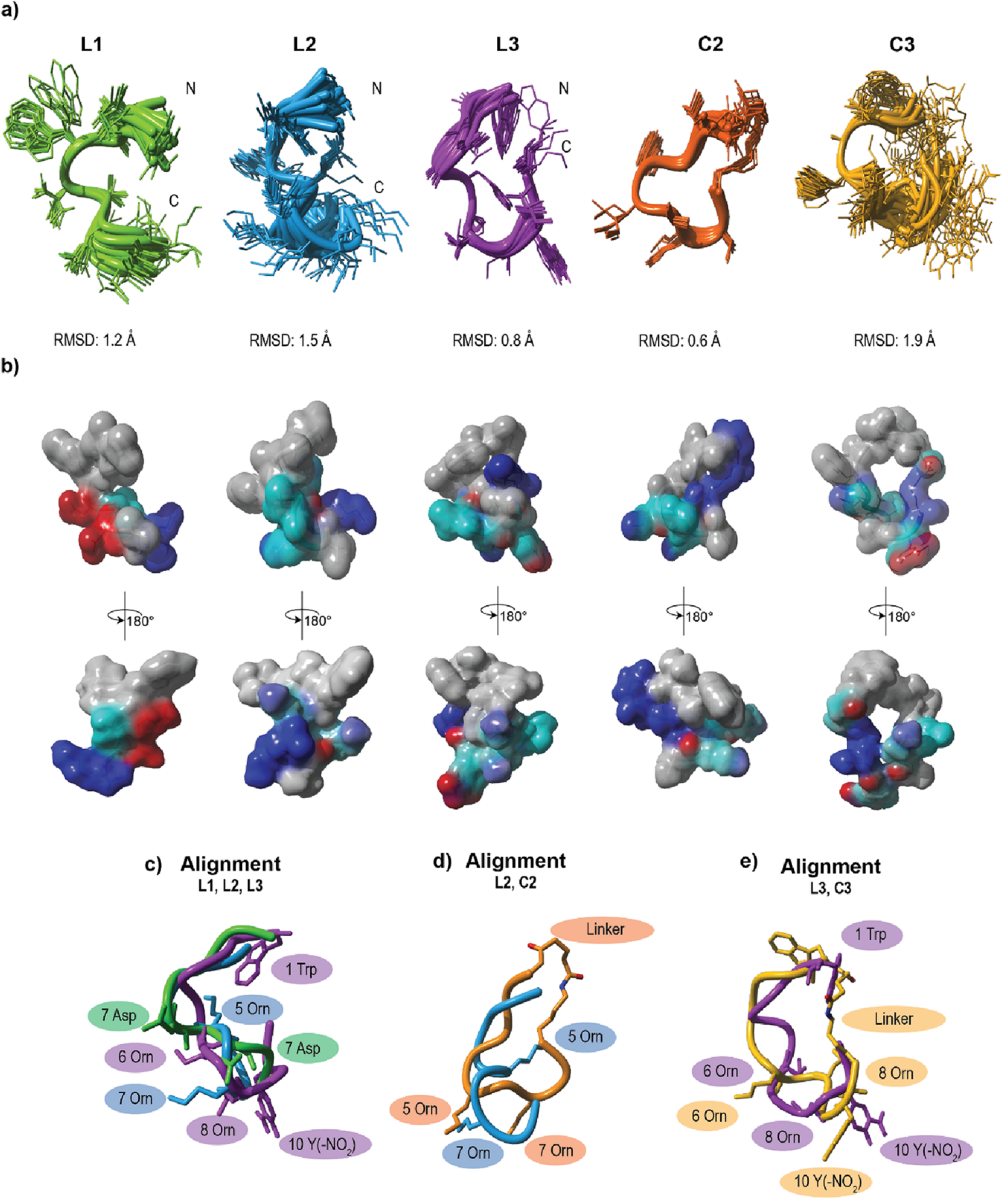
Solution NMR structures of peptides **L1**, **L2**, **L3**, **C2**, and **C3**. (a) Superposition of the NMR ensemble (20 lowest energy structures) and their respective backbone RMSD. (b) Surface representation (vander Waals, surface is colored according to the physicochemical properties of the amino acids: cyan – polar, blue – basic, red – acidic, gray – unpolar). (c) bb alignment of **L1** (green), **L2** (blue), and **L3** (purple). (d) bb alignment of **L2** (blue) and **C2** (orange). (e) bb alignment of **L3** (purple) and **C3** (yellow). Linker: glutaric anhydride.

To gain insight into the peptide’s possible mechanisms of action, the secondary structure of linear peptides **L1**, **L2**, and **L3** (Figure S12) was analyzed using CD spectroscopy, whereas NMR structure determination was conducted for **L1**, **L2**, **L3**, **C2**, and **C3** (Figure 5). The CD spectra of the three peptides do not resemble exactly the typical spectroscopic features of β-sheet, α-helical, or turn-harboring peptides (Figure S12). Despite the fact that the CD spectra of short peptides with unnatural amino acids are difficult to interpret, the shape of the absorbance and the absorbance maxima around 225 nm for peptides **L2** and **L3** seem to indicate a left-handed α-helix as it appears to be a mirror image of an α-helix containing peptides.^32^ However, the CD spectroscopic similarity between **L2** and **L3** clearly indicates some structural similarity (Figure S12). In contrast, the CD spectrum of **L1** did not indicate a defined secondary structure.

Henceforth, NMR spectroscopy was pursued for a more detailed structural analysis of the peptides. Linear peptides **L1**, **L2**, and **L3** possess a half-helix turn-like core, while the *C*- and *N*-termini remain unstructured and flexible (Figure 5a), as indicated by the ensemble backbone (bb) root-mean-square deviation (RMSD) ranging between 0.8 and 1.5 Å. Moreover, all peptides appear to be divided into a hydrophobic *N*-terminus and a more polar part at the *C*-terminus (Figure 5b).

Interestingly, peptide **C2**, which is the cyclic analog of peptide **L2**, rigidified significantly upon cyclization as indicated by a decrease of the bb RMSD from 1.5 to 0.6 Å and shows a well-defined structure (Figure 5a). In contrast, peptide **C3**, which is the cyclic counterpart for **L3** became more flexible, as reflected by the increased bb RMSD from 0.8 to 1.9 Å (Figure 5a). In parallel to the NOE-based structure analysis of the peptides, the temperature dependence of the NH chemical shifts was investigated to derive the temperature coefficients of the backbone NH protons to identify hydrogen bonds (Table S4). Two internal hydrogen bonds were found for peptide **L1** (Asp 5 and Thr 6) and one hydrogen bond for **C2** (Thr 6). With respect to the calculated NMR structure for peptide **L1**, Asp 5 most likely forms a hydrogen bond with its side chain, while Thr 6 forms a hydrogen bond with the amide oxygen of Ala 3. In the case of peptide **C2**, the hydrogen bond acceptor for the NH proton of Thr 6 is most likely the oxygen atom of the *N*-terminal amide, thus, partly responsible for its low flexibility.

Although, peptides **L1** and **L2** are structurally similar to **L3** (Figure 5c), it is **L3**, which shows potent antimicrobial activity. Furthermore, the cyclization of **L2** and **L3** resulted in a changed globular shape of the molecule (Figure 5c–e), which might be the main reason for the loss in activity of **C2** and **C3**.

### Bacterial Cytological Profiling

**Figure 6:**
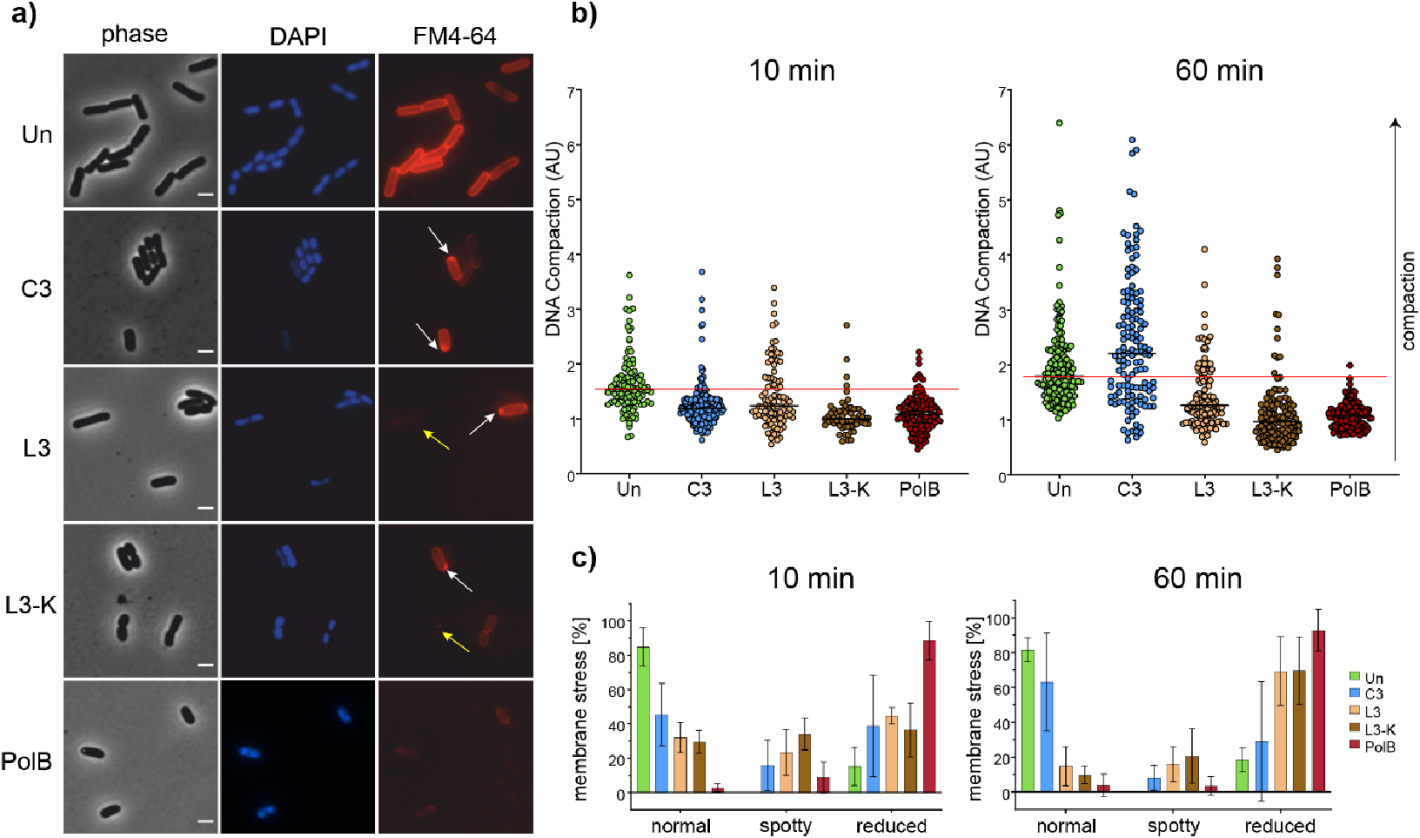
Bacterial cytological profiling of *E. coli*. (a) Fluorescence and phase contrast images of *E. coli* CCUG31246 stained with the red membrane dye FM4-64 and the blue DNA dye DAPI. Shown are images of cells treated with antibiotics for 60 min (see Figure S14 for corresponding images after 10 min). White arrows indicate cells with fluorescent membrane foci, while yellow arrows indicate cells with a reduced membrane staining (scale bar 2 μm). (b) Analysis of DNA compaction from microscopy images. Black lines indicate the median of each sample. The continuous red line marks the medial compaction value of the untreated control. Cells from three independent biological replicates were pooled for analysis. A minimum of 61 cells was analyzed per sample. (c) Quantification of membrane phenotypes from microscopy images. The bar chart shows the average of three independent biological replicates. Error bars represent the standard deviation of the mean. Cells with highly fluorescent membrane foci were counted as “spotty,” while cells with a visibly reduced or absent membrane stain were counted as “reduced.” A minimum of 140 cells was counted per sample. PolB: polymyxin B.

To gain insight into the peptides’ antimicrobial mechanisms, bacterial cytological profiling was performed. This live-cell imaging method makes use of different fluorescent dyes and protein fusions together with phase contrast microscopy to assess the phenotype of bacterial cells after antibiotic treatment.^33^ Single-cell analysis then gives insight into the extent and population heterogeneity of the observed phenotypic effects. In this study, we used the DNA dye DAPI and the membrane dye FM4-64 and analyzed the effects of the compounds on cell length, nucleoid compaction, and membrane morphology (Figures 6 and S15). To this end, *E. coli* CCUG31246 (uropathogenic clinical isolate) was chosen as a representative model and the peptides **L3**, **L3-K**, and **C3**, which showed activity against *E. coli* in the MMC assay, were tested. In the preparation of mechanistic studies, MICs were determined. For further experiments, 1× MIC was used for each peptide (64 μg/mL **L3**, 128 μg/mL **L3-K**, and 512 μg/mL **C3**). The lipopeptide polymyxin B, which permeabilizes both the inner and the outer membrane of Gram-negative bacteria, was used as a positive control (10 μg/mL). Cells were microscopically examined after 10 and 60 min of peptide treatment.

No marked effects were observed on cell length. Only **L3-K** showed very slightly shorter cells on average after 60 min of treatment (Figure S16). In contrast, the DAPI dye indicated that DNA compaction to be affected by all peptides (Figure 6a). Quantification of nucleoid compaction revealed that all peptides caused clear nucleoid relaxation after only 10 min of treatment (Figure 6b). This effect was even more apparent after 60 min for both **L3** and **L3-K**. This observation indicates that **C3**, **L3**, and **L3-K** affect DNA packing in a manner similar to polymyxin B. It should be noted that nucleoid relaxation is not a common phenotype caused by AMPs, e.g., tyrocidines and gramicidin S have been shown to have the opposite effect on bacteria.^34^ Interestingly, **C3** displayed a considerable population heterogeneity after 60 min of treatment, showing individual cells with normal, relaxed, and condensed nucleoids. This could be indicative of cells in different stages of inhibition or due to different reactions of individual cells of an inherently heterogeneous bacterial population.

Clear effects were also observed on the bacterial membrane morphology in the FM4-64 stain (Figure 6a). All three peptides showed two subpopulations with distinct phenotypes: those with strongly fluorescent membrane foci (white arrows, Figure 6a) and those where membrane staining was reduced or not visible at all (yellow arrow, Figure 6a). Most membrane dyes, including FM4-64, prefer more fluid membrane regions and accumulate in those areas when phase separation occurs, appearing as intensely fluorescent foci. Conversely, membrane dyes are often depleted from rigid membrane regions or show less intense fluorescence in rigid membrane environments.^35,36^ Thus, our results point to membrane phase separation in cells with bright foci and possibly increased membrane rigidity in cells with a very weak membrane stain. When quantifying these phenotypes, a clear trend toward a higher proportion of cells with reduced membrane staining at 60 min compared to 10 min became apparent (Figure 6c), suggesting a two-stage effect, where cells first undergo a transient membrane phase separation, possibly followed by overall membrane rigidification. However, it must be noted that FM4-64 binds to both the inner and the outer membrane of Gram-negative bacteria, depending on inner membrane accessibility,^37,38^ and thus, does not allow reliable distinction between inner and outer membrane effects. The positive control polymyxin B displayed the same distinct phenotypes, yet over 90% of cells displayed the unstained phenotype already after 10 min, suggesting that it acts much faster than **L3** and **L3-K**. In line with the DAPI results, **C3** showed considerable population heterogeneity as well as sample-to-sample variation in the FM4-64 stain, which is reflected by the high error bars in Figure 6c.

**Figure 7:**
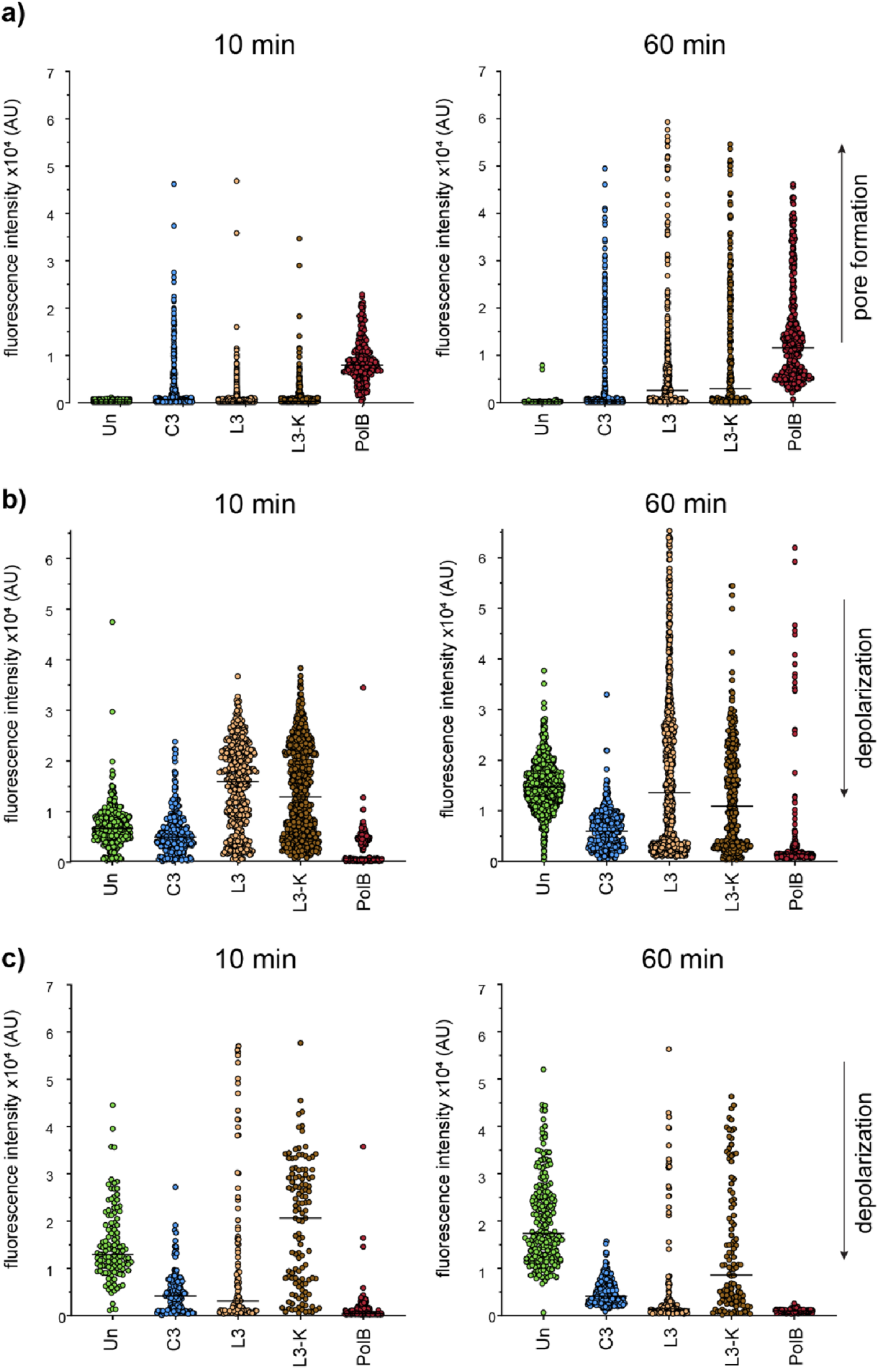
Membrane permeability of *E. coli* upon exposure to peptides **C3**, **L3**, **L3-K**. (a) Uptake of propidium iodide by *E. coli* CCUG31246 treated with C3, L3, L3-K, and PolB for 10 and 60 min. (b) DiSC(3)5 measurements of *E. coli* CCUG31246 treated with C3, L3, L3-K, and PolB for 10 and 60 min. Black lines indicate the median of each sample. (c) DiSC(3)5 measurements of *E. coli* MC4100 pABCON2-fhuA ΔC/Δ4L (outer membrane permeable through overexpression of the porin FhuA) treated with C3, L3, L3-K, and PolB for 10 and 60 min. Black lines indicate the median of each sample. Cells from three independent biological replicates were pooled for analysis. A minimum of 114 cells was analyzed per sample. PolB: polymyxin B.

Due to the overall similarity of the peptides’ cytological profiles to that of polymyxin B, we further tested their ability to form pores in the cell membrane. To this end, we used the fluorescence probe propidium iodide, which cannot cross intact membranes but can enter cells through pores of sufficient size.^39^ Pore-forming peptides, such as polymyxin B, lead to near-instantaneous uptake of the dye throughout the bacterial population. This effect was indeed observed here with polymyxin B, but only a small subpopulation of cells treated with **C3**, **L3**, and **L3-K** showed increased fluorescence (Figure 7a), suggesting that the peptides do not act by pore formation. While the proportion of fluorescent cells increased after 60 min, a high number of cells (31% for **L3**, 35% for **L3-K**, 64% for **C3**) remained unstained, indicating that the single red-stained cells are most likely perforated due to undergoing cell lysis as a consequence of peptide-induced cell death.

Propidium iodide is a large organic molecule that is not suitable to detect smaller ion-conducting pores or channels induced by antimicrobials. To assess whether the peptides may form smaller membrane pores sufficient for the passage of ions, we tested their effects on the membrane potential using the fluorescence probe DiSC(3)5.^40^ This dye accumulates in the cell membrane in a membrane potential-dependent manner and disassociates, when the membrane potential is dissipated. If a pore, large or small, is formed in the cell membrane, a clear and immediate reduction in the DiSC(3)5 fluorescence intensity is observed. This effect can be clearly seen with polymyxin B (Figure 7b). In contrast, **C3** had no effect on the membrane potential after 10 min and caused partial depolarization after 60 min. **L3** and **L3-K** displayed an increased fluorescence signal at 10 min and a heterogeneous population of partially depolarized cells and cells with a higher fluorescence signal after 60 min.

This behavior may be indicative of outer membrane permeabilization, as DiSC(3)5 has only limited outer membrane permeability, and its uptake is increased when the outer membrane is permeabilized, resulting in a heterogeneous cell population with an overall higher fluorescence signal. This effect is, for example, observed after short treatment times with polymyxin B (data not shown). It is conceivable that **L3** and **L3-K** similarly permeabilize the outer membrane, yet at a much slower time scale. To test this hypothesis, we employed an *E. coli* strain that overexpresses the outer membrane porin FhuA, making these cells more permeable to most fluorescence dyes including DiSC(3)5.^41^ Indeed, the consecutive increase and decrease of fluorescence intensity observed in the wild type was strongly reduced in the FhuA-overexpressing strain (Figure 7c). This observation suggests that **L3** and **L3-K** first permeabilize the outer and then the inner membrane. Interestingly, in the FhuA-overexpressing strain, **L3** completely depolarized most cells after 10 min, while the effect of **L3-K** only set in after 60 min and remained heterogeneous. This shows that these two peptides in principle have very different inner membrane permeabilization kinetics. These do not become apparent in the wild type, where the outer membrane is fully intact, yet it will be interesting to take into consideration when these qualified hit structures will be modified for future drug development.

**C3** behaved similarly in both *E. coli* strains, showing stronger depolarization in the FhuA-overexpressing strain, which suggests that it is partially retained by the outer membrane. This effect together with the absence of highly fluorescent cells in the wild-type samples suggests that, in contrast to **L3** and **L3-K**, this peptide does not notably permeabilize the outer membrane.

Taken together, our data show that **L3** and **L3-K** affect both the outer membrane and inner membrane of Gram-negative bacteria. They do not form pores large enough for efficient uptake of the propidium iodide probe but allow the passage of small ions, resulting in dissipation of the membrane potential. Thereby, they act on a much slower scale than polymyxin B and do not cause complete membrane depolarization. Since a pore would cause immediate and complete depolarization, we can conclude that **L3** and **L3-K** instead slowly increase the passive permeability of the cell membrane. Together with our FM4-64 stain, we can hypothesize that this may be due to phase boundary defects caused by membrane phase separation.

**C3** showed similar effects on membrane phase separation. It did not show any effect on the outer membrane and had only mild effects on the membrane potential. These differences suggest that **C3** probably acts similarly to **L3** and **L3-K** but is specific for the inner membrane, while the other two peptides display a dual activity on both membranes of *E. coli*.

All three peptides cause relaxation of the nucleoid, which is indicative of DNA packing defects. The same effects were observed for polymyxin B, suggesting that this could be a yet unknown consequence of their interaction with the inner membrane. However, this is not a general effect of membrane-targeting antimicrobial peptides, and an additional independent mechanism, possibly involving peptide translocation into the cytosol and interaction with DNA, cannot be excluded at this stage.

## Conclusions

Based on genome analysis of *Streptomyces* sp. HKF8 isolated from a Northern Chilean Patagonia fjord and computational prediction by antiSMASH as well as secondary metabolite profiling, linear and cyclic peptides have been synthesized and studied toward their microbicidal activity, structural properties, and mechanisms of action. Our results show that though sequentially similar, the peptides show different microbicidal and structural properties. Peptide **L3** showed the best killing activity for *S. aureus*, *E. coli*, and *C. albicans* in a range from 6.3 to 12.5 μg/mL. Interestingly, removing the basic amino acid Lys from the *C*-terminus (**L3-K**) and, therefore, decreasing the overall charge of the peptide resulted in a slight loss of microbicidal activity, while cyclization (**C3**) had a dramatic effect on microbicidal activity. Cyclization of the peptide **L3** also had a large impact on its backbone stability. Surprisingly, backbone RMSD increased from 0.8 Å for linear peptide **L3** to 1.9 Å for its cyclic counterpart **C3**. Generally, NMR structure analysis and determination revealed, that the peptides possess a half-helix turn-like core, while the *C*- and *N*-termini remain unstructured and flexible. First insights into the peptides’ mechanisms of action by performing bacterial cytological profiling using uropathogenic *E. coli* as model indicate that the most active peptides **L3** and **L3-K** affect both the outer and inner membrane of Gram-negative bacteria. They do not form pores large enough for efficient uptake of the fluorescence probe propidium iodide. While both peptides allowed the passage of smaller ions, eventually resulting in dissipation of the membrane potential, only **L3** showed this effect after 10 min while **L3-K** still did not cause complete depolarization at 60 min, showing that at least the latter cannot act through similar formation of ion-conducting pores. Furthermore, both peptides cause relaxation of the nucleoid, which is indicative of DNA packing defects. However, at this stage, this effect cannot yet be ascribed to either a consequence of their membrane interaction or an independent secondary activity.

Taken together, this study represents a promising strategy to discover unknown serum stable and noncytotoxic antimicrobial qualified hit structures from marine terrain with novel modes of action, which can be a good starting point toward finding new core structures from previously unexplored natural sources.

## Methods

### Genome Mining of *Streptomyces* sp. H-KF8

The BGCs of *Streptomyces* sp. H-KF8 were identified through the online platform AntiSMASH (version 6.0),^25^ using the genome.^21^ Genetic determinants and peptidic prediction were manually validated.

### Peptide Synthesis

All peptides were synthesized by automated Fmoc-based SPPS on an INTAVIS MultiPep synthesizer. Peptides were synthesized on Rink amide resin with a loading capacity of 0.54 mmol/g. All amino acid derivatives were prepared as stock solutions at a concentration of 0.5 M. All derivatives were dissolved in DMF and mixed vigorously in a vortex until complete solubilization. Hexafluorophosphate benzotriazole tetramethyl uronium (HBTU) was used as the coupling reagent and prepared as 0.5 M stock solution (5 equivalents relative to loading capacity) in DMF, and NMM (two-fold excess to amino acids and coupling reagent, 10 equivalents relative to loading capacity) stock solution in DMF was used as a base. Fmoc-deprotection was performed with 20% piperidine in DMF. Coupling of each amino acid was performed twice each time for 20 min, as well as Fmoc-deprotection each time for 10 min. NMP was used as a cosolvent during coupling. After every coupling, DMF washing was performed. Peptide cleavage and deprotection were accomplished in a mixture of 92.5% TFA, 5% water, and 2.5% TIPS.

### Synthesis of Cyclic Peptides

All cyclic peptides were synthesized by on-resin cyclization. The peptides were first synthesized as linear peptides and, after completion, subjected to an on-resin cyclization procedure directly after the last coupled amino acid. The procedure started with resin washing with *N*-methyl-2-pyrrolidone (NMP), followed by anhydride coupling to the *N*-terminal amino group. For the latter, a mixture of glutaric acid anhydride:DMAP:DIEA (10:1:10) in NMP (0.2 mL per eq.) was incubated for 1 h at 75 °C. After that resin was washed intensively three times each with NMP followed by washing three times with DCM. Then, monomethoxytrityl (Mmt) was cleaved from the lysine side chain selectively with the mixture containing 1%TFA, 5%TIPS, and 94% DCM for 5 min twice by discarding cleavage solution each time. The resin was finally washed intensively with DCM for five times. The head-to-tail amide bond cyclization was performed by using different coupling reagents **C1** (5 eq. pyBOP and 10 eq. DIEA), **C2** (5 eq. HATU and 10 eq. NMM), and **C3** (1.5 eq. pyBOP and 3.3 eq. TMP). The coupling was performed twice for 2 h at 75 °C. After the cyclization thorough washing with DMF and DCM, the resin was dried before cleavage.

### High-Performance Liquid Chromatography (RP-HPLC)

All crude and purified peptides were analyzed by analytical RP-HPLC on a Waters e2695 Alliance system (Waters, Milford, MA, USA) employing a Waters 2998 photo diode array (PDA) detector equipped with an ISAspher Xela 100-1.7 C18 column (50 × 2.1 mm). HPLC eluent A was water (0.1% trifluoroacetic acid (TFA)) and eluent B was acetonitrile (0.1% TFA) (detection at 214 nm).

Preparative scale purification of the peptides was achieved employing a Waters 1525 binary gradient pump and a Waters 2998 PDA detector or a customized Waters 600 module equipped with a Waters 996 PDA detector (Waters). HPLC eluent A was water (0.1% TFA), and eluent B was acetonitrile (0.1% TFA).

### Mass Spectrometry

Bioactive fraction obtained from crude extracts of *Streptomyces* sp. H-KF8 was identified with tests against *Staphylococcus aureus* ATCC 29740T, *Staphylococcus epidermidis* ATCC 35984T, *Escherichia coli* ATCC 8739T, *Listeria monocytogenes* ATCC 19114T, *Pseudomonas aeruginosa* ATCC 27853T, *Klebsiella pneumoniae* ATCC 13883T, *Enterococcus faecalis* ATCC 19433T, *Micrococcus luteus* ATCC 9341T, and *Bacillus subtilis* ATCC 1668T, and bioactive fractions were subjected to ESI-FT ICR MS analysis. For initial prediction of the consensus sequence from *Streptomyces* sp. H-KF8, MALDI-TOF MS/MS analysis was performed by Bruker Ultraflex Extreme spectrometer, samples were prepared using α-cyano-4-hydroxycinnamic acid and measured in positive mode. The parent peak at *m*/*z* 1448.752 was selected for MS/MS analysis (Figures S1 and S2).

The molecular weight of the purified compounds was confirmed by ESI mass spectrometry on a Waters SYNAPT G2-Si HD-MS spectrometer equipped with a Waters Acquity UPLC system (Waters). Leu-enkephalin was used as a reference compound for high-resolution measurements.

### Amino Acid Analysis

To determine the concentration (calculated as active peptide content according to a given sample mass), amino acid analysis was performed as previously reported.^42^ (Table S3 and Figure S11).

### NMR

Solution NMR experiments were performed at 280 K on a Bruker Avance III HD 900 MHz spectrometer. The peptide sample was dissolved in 90% H_2_O/10% D_2_O using the freeze-dried solid compound. Data were acquired and processed with Topspin 4.1.3 (Bruker, Rheinstetten, Germany) and analyzed with CCPN.^43^ The proton resonance assignment was performed using a combination of 2D [1H,1H]-TOCSY (80 ms spinlock time) and [1H,1H]-NOESY and/or [1H,1H]-ROESY experiments. Distance constraints were extracted from [1H,1H]-NOESY and [1H,1H]-ROESY spectra acquired with 200–300 ms mixing time. The upper limit distance constraints were calibrated according to their intensity in the NOESY/ROESY spectrum. Torsion angle constraints were obtained from proton chemical shift analysis using DANGLE^44^ and adapted accordingly to d- and l-amino acids.^45^ Structure calculations were performed with the YASARA structure (Table S5).^46−48^ Structures were refined in water at pH 4.

### CD Spectroscopy

Circular dichroism (CD) spectra were recorded on a Chirascan Applied Photophysics in a cuvette with a length of 1 cm between 190 and 280 nm and a bandwidth of 1.0 nm with a step size of 1 nm and a response time of 7.6 s per point. CD spectra were recorded as absorbance in mdeg at a temperature of 23 °C. The background was measured and subtracted from the CD spectra from the samples by measuring a blank sample with the corresponding solvent. Peptides were dissolved in two different concentrations (10 μM and 50 μM) in water.

### Minimum Microbicidal Concentration (MMC_99_)

The microbicidal activity of the synthetic peptides was assessed against *S. aureus* (ATCC 12600; American Type Culture Collection, Manassas, VA, USA), *Escherichia coli* (CCUG 31246; Culture Collection, University of Gothenburg, Sweden), and *Candida albicans* (ATCC 64549/CCUG31028) using an MMC assay, as described previously by Haversen et al. (specific details in Supporting Information, pages 10, 11).^28^ The assay was performed in BHI diluted 1/100 (BHI_100_). Two-fold dilution series were performed on all peptides, starting at 100 μg/mL down to 1.6 μg/mL. Fusidic acid, polymyxin B (PolB), and clotrimazole were used for comparison.

### Strains and Growth Conditions for Mechanism of Action Studies

*E. coli* CCUG31246 and MC4100 (F–-(*araD139*) Δ(*argF-lac*)169 λ–e14–*flhD5301* Δ(*fruK-yeiR*)725(*fruA25*) *relA1 rpsL150*(Strr) *rbsR22* Δ(*fimB-fimE*)632(:IS1) *deoC1*, *spoT1*)^49^ carrying pABCON2-*fhuA* ΔC/Δ4L^41,50^ were aerobically grown at 37 °C in 1:10 diluted BHI.

### Minimal Inhibitory Concentration

MICs were performed under the same growth conditions used for mode of action analysis following the guidelines issued by the Clinical Laboratory Standardization Institute (CLSI) with slight modifications. Serial two-fold dilutions of the peptides were prepared in 1:10 BHI in a sterile 96-well plate and subsequently inoculated with 5 × 10^5^ CFU/mL of *E. coli* CCUG31246. MIC plates were incubated at 37 °C for 16 h under steady agitation. The following MICs were obtained: **C3**: 512 μg/mL, **L3**: 64 μg/mL, and **L3-K**: 128 μg/mL. These concentrations were used for all mechanistic experiments. Polymyxin B was used as a positive control in all assays at a final concentration of 10 μg/mL.

### Peptide Stability in Serum

Peptide serum stability was performed according to D́Aloisio et al.^30^ and Chen et al.^51^ with some minor modifications. The serum stability was investigated using human serum (Sigma-Aldrich) from Human male AB plasma, USA origin, sterile-filtered. 250 μL serum was temperature-equilibrated at 37 °C and 100 μL aqueous peptide solution (**L2**: 1 mM; **L3**: 0.9 mM; **L3-K**: 0.7 mM; and **C3**: 0.5 mM) was added. RP-HPLC was measured at time intervals of 0, 0.5, 1, 4, and 24 h. For RP-HPLC samples, 30 μL of peptide solution was taken, 7 μL of Fmoc-Gly solution (4 mM) was added, and 2 μL of the sample was injected to HPLC. Fmoc-Gly was used as an internal standard (RP-HPLC chromatograms are shown in Figure S13).

### Hemolysis

The hemolysis assay was performed according to Myhrman et al.^52^ with some minor modifications. In short, the hemolytic activities of the peptides **L2**, **L3**, **L3-K**, and **C3** were determined using fresh human erythrocytes from blood donors. The erythrocytes were separated from blood supplemented with EDTA by centrifugation at 1000*g* for 5 min, washed three times with PBS (pH 7.4), and resuspended in PBS to a final red blood cell (RBC) concentration of 2% (v/v). The peptide was serially diluted by two-fold steps in PBS in 80 μL volumes (in triplicates) in a round-bottom 96-well plate (Sarstedt, Numbrecht, Germany, 82.1582001). An equal volume of the RBC suspension was added to the wells, and the plate was incubated for 1 h at 37 °C. After the incubation, 100 μL of the supernatants was carefully removed and transferred to a new microplate. Since **L3**, **L3-K**, and **C3** turned yellow in PBS, which increased the absorbance, the pH was adjusted to 5 in all transferred supernatants. At this pH, the yellow color of the peptides disappeared, while the color of hemoglobin of the positive control was unaffected at 490 nm. The release of hemoglobin was analyzed by measuring the absorbance of the supernatants at 490 nm (minus 650 nm) (Tecan). The negative control consisted of PBS instead of peptide, and the positive control consisted of 0.1% Triton X-100 (total hemolysis). The percentage of hemolysis was calculated for all transferred samples using the abs_490–650_ of the Triton X-100 containing sample as 100% hemolysis.

### Cytotoxicity

Cytotoxicity against human embryonic kidney (HEK) and hepatoblastoma (HepG2) cell lines was assessed. Cells were grown to an initial seeding density of 10 000 cells per well. Metabolic activity was determined in a resazurin-based assay. HEK and HepG2 cells were exposed to four different concentrations of the peptides for 24 h. Treatment with Triton X-100 at 1% v/v was used as a positive control. Following this exposure period, the cells were treated with resazurin at a final concentration of 0.015 mg/mL for 3 h. The metabolic activity was then determined in an resazurin-based assay. The fluorescence intensities were quantified in a Hidex Sense microplate reader using an excitation wavelength of 544 nm and an emission wavelength of 590 nm.

### Bacterial Cytological Profiling

Bacterial cytological profiling was performed according to Wenzel et al.^34^ In short, *E. coli* CCUG31246 was grown until an OD_600_ of 0.3 before antibiotic addition. Samples were taken after 5 and 55 min of antibiotic treatment and subsequently stained with 0.5 μg/mL FM4-64 (Invitrogen) and 1 μg/mL DAPI (Invitrogen) for an additional 5 min. Stained samples were spotted on 1.2% agarose films, sealed with a gene frame, and immediately imaged using a Nikon Eclipse Ti2 inverted fluorescence live-cell imaging system equipped with a CFI Plan Apochromat DM Lambda 100X Oil objective (N.A. = 1.45 and W.D. = 0.13 mm), a Photometrics, PRIME BSI camera, a Lumencor Sola SE II FISH 365 light source, and an Okolab temperature incubation chamber. Images were obtained using the NIS elements AR software version 5.21.03 and were processed and analyzed with ImageJ.^53^ Quantification of microscopy images was performed using the ImageJ plugins ObjectJ^54^ and MicrobeJ.^55^

Cell length was analyzed based on phase contrast images in ObjectJ using default parameters.^54^ DNA compaction was analyzed using MicrobeJ^55^ based on DAPI and phase contrast images. The parameters for cell and DNA recognition were set to default parameters. The area and width were adjusted to the minimal measured cell length of each sample to ensure detection of all bacterial cells while reducing false-positive detection of debris. Fluorescence intensity parameters remained at default settings. The *Z*-score was adjusted manually to ensure fitting of DNA detection. DNA compaction values were derived from the quotient of the cell area divided by the DNA area.

### Propidium Iodide Staining

Pore formation was investigated with the fluorescence dye propidium iodide as described previously^56^ with minor modifications. *E. coli* CCUG31246 was grown to an OD_600_ of 0.3 and subsequently treated with different peptides for 10 and 60 min. Samples were stained with 1 μg/mL propidium iodide for 15 min (added 5 min before adding antibiotics for the 10 min time point and after 45 min of antibiotic treatment for the 60 min time point), spotted on 1.2% agarose films, and sealed with a gene frame. Microscopy was performed as described above. The fluorescence intensity of the different samples was analyzed with MicrobeJ. For detection of bacterial cells from phase contrast images, parameters were set to an area of 1.5-max, length of 1-max, width 0.5–2.5, curvature 0–1.5, and an angularity of 0–0.5. Fluorescence intensity parameters were set to an area of 1.5-max, length of 1-max, and width 0.5–2.5. All other parameters remained at default settings.

### DiSC(3)5 Microscopy

The membrane potential of *E. coli* CCUG31246 and *E. coli* MC4100 carrying pABCON2-*fhuA* ΔC/Δ4L was measured by DiSC(3)5 microscopy according to ref. (40) with minor modifications. Cells were grown in 1:10 BHI containing 50 μg/mL bovine serum albumin to an OD_600_ of 0.3 prior to antibiotic treatment with the respective peptides for 10 and 60 min. Samples were stained with 0.5 μM DiSC(3)5 for 15 min (added 5 min before adding antibiotics for the 10 min time point and after 45 min of antibiotic treatment for the 60 min time point). Stained samples were spotted on 1.2% agarose, sealed with a gene frame, and imaged immediately. Microscopy was performed as described above, and fluorescence intensity was analyzed with the same parameters used for propidium iodide detection.

## Supporting Information Available

Peptide from *Streptomyces* sp. H-KF8 (Figures S1, S2 and Table S1), HPLC and mass spectrometry (Table S2 and Figures S3–S10), amino acid analysis (Figure S11 and Table S3), circular dichroism spectroscopy (Figure S12), NMR analysis (Tables S4 and S5), MMC_99_ activity including salt stability measurements (Table S6), serum stability RP-HPLC chromatograms (Figure S13), hemolysis (Figure S14), and bacterial cytological profiling of peptides (Figures S15 and S16) (PDF)

## Abbreviations

DMAP: 4-dimethylaminopyridine
DIEA: *N*,*N*-diisopropylethylamine
HATU: 1-[bis(dimethylamino)methylene]-1*H*-1,2,3-triazolo[4,5-*b*]pyridinium 3-oxide hexafluorophosphate
TMP: 2,2,6,6-tetramethylpiperidine
NMM: *N*-methylmorpholine
pyBOP: (benzotriazol-1-yloxy)tripyrrolidinophosphonium hexafluorophosphate
BHI: brain heart infusion
N.A.: numerical aperture
W.D.: working distance
