# Supplementary material for: Mimicking Nonribosomal Peptides from the Marine Actinomycete Streptomyces sp. H-KF8 Leads to Antimicrobial Peptides
Source: ACS Infect Dis. 2023 Dec 19;10(1):79–92. doi: 10.1021/acsinfecdis.3c00206 (PMC10788856; doi:10.1021/acsinfecdis.3c00206)
Supplement: Supplementary file 1 — id3c00206_si_001.pdf [file id3c00206_si_001.pdf]

Supporting Information

Mimicking Non-ribosomal Peptides from the Marine Actinomycete *Streptomyces* sp. H-KF8 Leads to Antimicrobial Peptides.

Luisa I. Beyer,[a, c] Ann-Britt Schäfer,[b, c] Agustina Undabarrena,[d] Inger Mattsby-Baltzer,[e] Daniel Tietze,[a, c] Elin Svensson,[b] Alexandra Stubelius,[b] Michaela Wenzel,[b, c] Beatriz Cámara,[d] and Alesia A. Tietze,*[a, c]

a University of Gothenburg, Department of Chemistry and Molecular Biology, Wallenberg Centre for Molecular and Translational Medicine, Medicinaregatan 7B, 413 90 Gothenburg, Sweden, E-mail: alesia.a.tietze@gu.se

b Chalmers University of Technology, Department of Life Sciences, Kemigården 4, 412 96 Göteborg, Sweden

c Center for Antibiotic Resistance Research in Gothenburg, The University of Gothenburg, Box 100, 405 30 Göteborg, Sweden

d Universidad Técnica Federico Santa María, Departamento de Química & Centro de Biotecnología Daniel Alkalay Lowitt, Laboratorio de Microbiología Molecular y Biotecnología Ambiental, Valparaíso 2340000, Chile

e University of Gothenburg, Department of Infectious Diseases, Institute of Biomedicine, The Sahlgrenska Academy at University of Gothenburg, Box 440, 405 30 Göteborg, Sweden

Table of content

Peptide from *Streptomyces* sp. H-KF8 ..... S2
HPLC and Mass spectrometry S3
Amino acid analysis S8
HPLC for linear calibration S8
Calculation of peptide content S8
Circular dichroism spectroscopy S9
NMR S9
MMC_{99} activity including salt stability measurements S11
Serum stability S12
Hemolysis S13
Bacterial cytological profiling of peptides S13

Peptide from *Streptomyces* sp. H-KF8

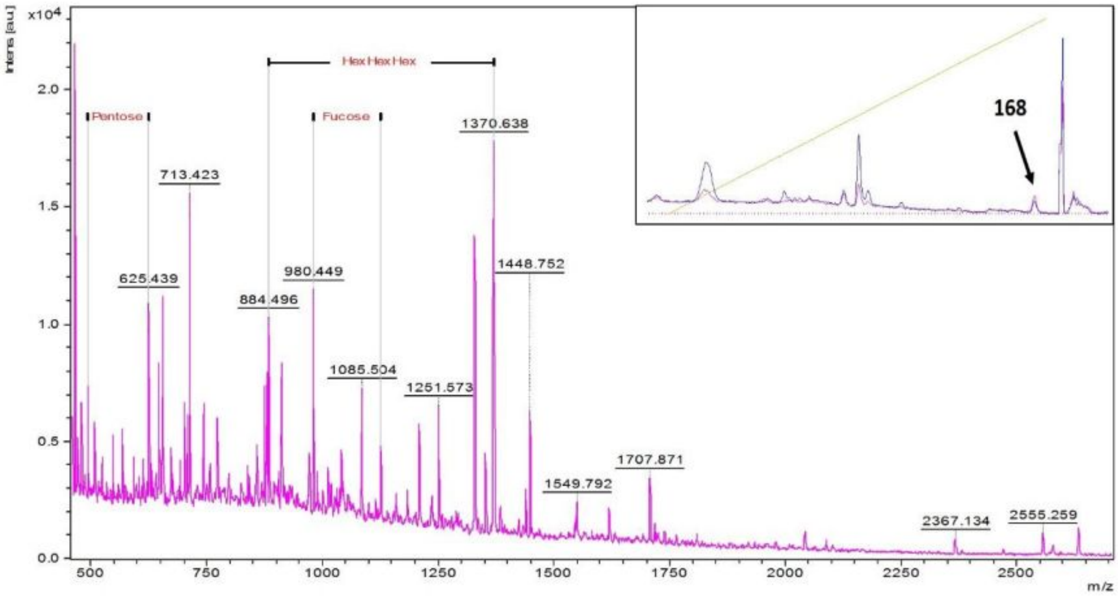

Figure S1. ESI-FT ICR MS mass spectrum for fraction 168 obtained from *Streptomyces* sp. H-KF8 grown in ISP2 medium. Inset: HPLC chromatogram depicting bioactive fraction (No 168).

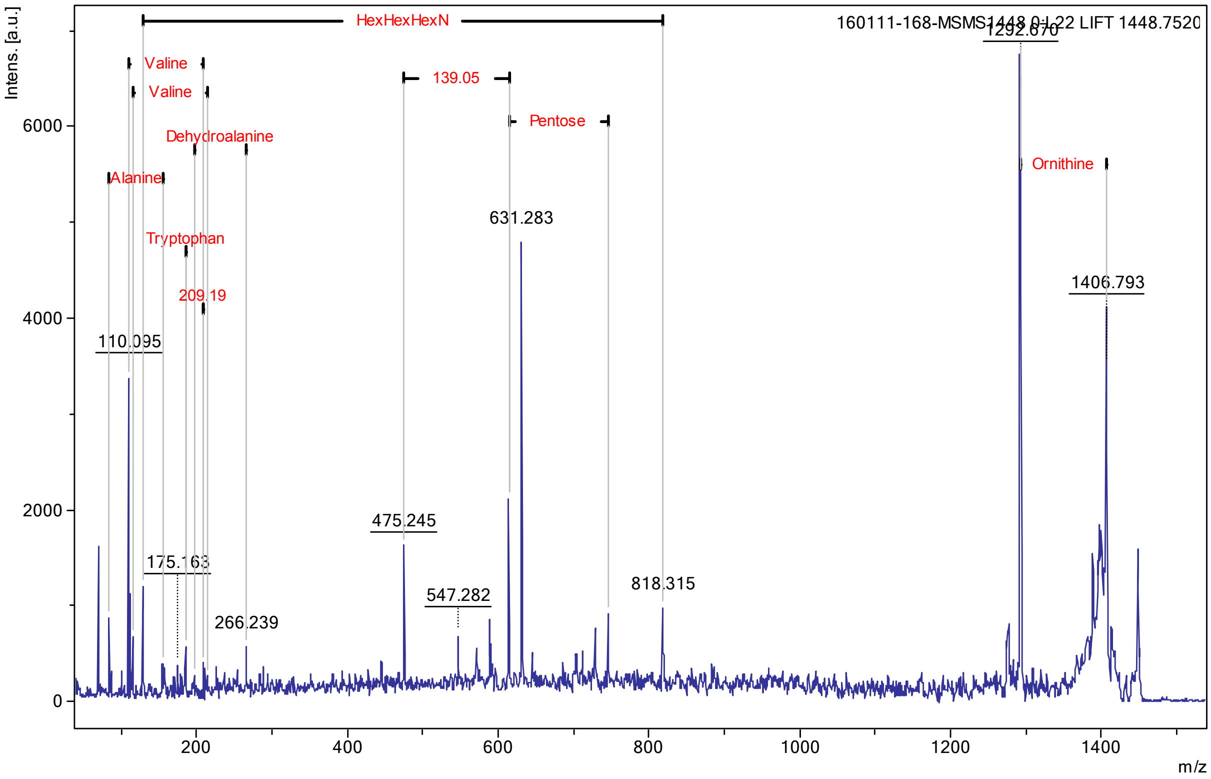

Figure S2. MALDI TOF MS/MS *Streptomyces* sp. H-KF8 parent peak m/z 1448.752.

Table S1. Genome-based predictions of NRPS #1.8 BGC of *Streptomyces* sp. H-KF8.

Gene N	Gene Category	Gene Predicted Function*
1	Inmunity	Glycopeptide resistance protein (VanZ family)
2	Regulators	Serine phospatase protein (SpoIIE familiy)
3	Other	AAA+ domain containing protein (Chaperone family)
4	Other	Hypothetical protein
5	Tailouring enzymes	Diiron oxigenase (AurF family)
6	Other	4Fe-4S dicluster-domain containing protein
7	Other	Hypothetical protein
8	Regulators	Serine phospatase protein (SpoIIE familiy)
9	Other	Hypothetical protein
10	Biosynthesis	Thioesterase
11	Tailouring enzymes	Cytocrhome P450
12	Inmunity	Serine hydrolase β-lactamase
13	Biosynthesis	NRPS
14	Biosynthesis	NRPS
15	Biosynthesis	MbtH family protein
16	Tailouring enzymes	N-actyltransferase protein (GNAT family)
17	Other	Hypothetical protein
18	Tailouring enzymes	Neuramidase
19	Tailouring enzymes	Sugar O-acetyltransferase
20	Tailouring enzymes	B-galactosidase
21	Inmunity	Antibiotic resistance protein (VOC family)
22	Inmunity	Metallopeptisase protein (M15 family)
23	Tailouring enzymes	N-acetyltransferase protein (GNAT family)
24	Regulators	Transcriptional regulator (LacI family)
25	Transporters	Sugar ABC transporter
26	Transporters	Sugar ABC transporter permease
27	Transporters	Sugar ABC transporter permease
28	Tailouring enzymes	B-glucosidase
29	Other	DUF397 domain-containing protein
30	Regulators	Two component system (DNA-binding domain)
31	Regulators	Two component system (histidine kinase domain)

*Based on BLASTp results

HPLC and Mass spectrometry

Table S2. Analytical data for synthesized peptides.

Name	Peptide	Sum formula	Calculated MW* [g/mol]	Experimental MW* [g/mol]	Δppm
**L1**	$_{D}A_{D}V_{D}AW_{D}DT_{D}D_{D}VK$-NH2	$C_{45}H_{70}N_{12}O_{14}$	502.2646	502.2660	2.79
**C1**	cyclo[(CO)C3H6CO$_{D}A_{D}V_{D}AW_{D}DT_{D}D_{D}VK$] -NH2	$C_{50}H_{74}N_{12}O_{16}$	550.2751	550.2740	2.00
**L2**	$_{D}A_{D}V_{D}$AWOrnTOrn$_{D}$VK-NH2	$C_{47}H_{80}N_{14}O_{10}$	501.3169	501.3175	1.20
**L2-K**	$_{D}A_{D}V_{D}$AWOrnTOrn$_{D}$V-NH2	$C_{41}H_{68}N_{12}O_{9}$	437.2694	437.2675	4.35
**C2**	cyclo[(CO)C3H6CO$_{D}A_{D}V_{D}$AWOrnTOrn$_{D}$V K]-NH2	$C_{52}H_{84}N_{14}O_{12}$	549.3275	549.3272	0.55
**L3**	W$_{D}$A$_{D}$V$_{D}$AWOrnTOrn$_{D}$V(Y-NO2)K]-NH2	$C_{67}H_{98}N_{18}O_{15}$	698.3808	698.3826	2.58
**L3-K**	W$_{D}$A$_{D}$V$_{D}$AWOrnTOrn$_{D}$V(Y-NO2)]-NH2	$C_{61}H_{86}N_{16}O_{14}$	634.3333	634.3363	4.73
**C3**	cyclo[(CO)C3H6COW$_{D}$A$_{D}$V$_{D}$AWOrnTOrn $_{D}$V(Y-NO2)K]-NH2	$C_{72}H_{102}N_{18}O_{17}$	746.3914	746.3895	2.55

* Mass peaks detected as $[M+2H]^{2+}$

L1

(I) **(II)**

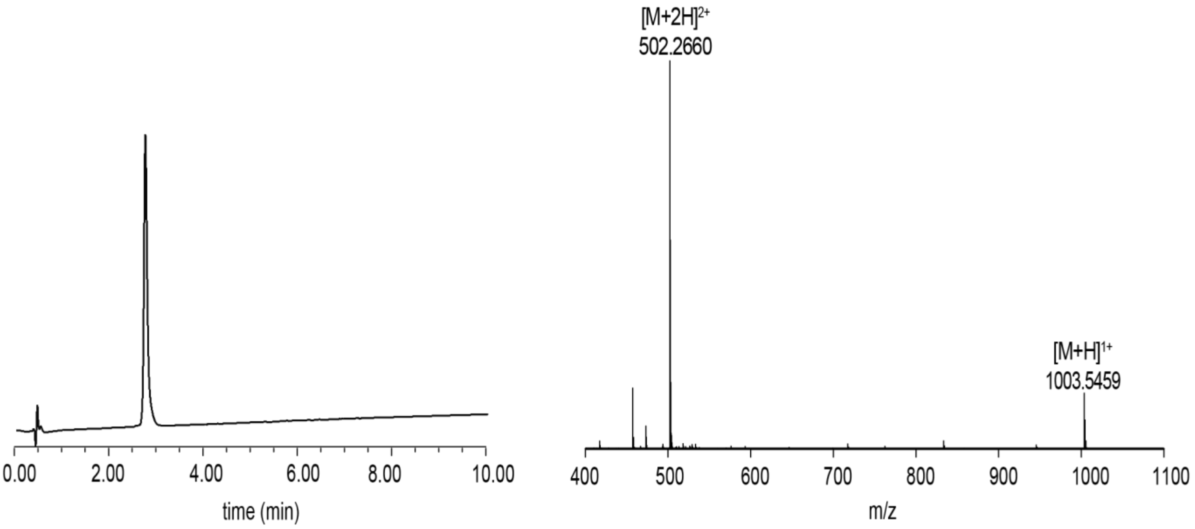

Figure S3. (I) RP-HPLC chromatogram (gradient from 10 to 50% acetonitrile in water over 10 min at 2 ml/min, detection at 214 nm) and (II) high resolution mass spectrum for peptide **L1**.

L2

(I) **(II)**

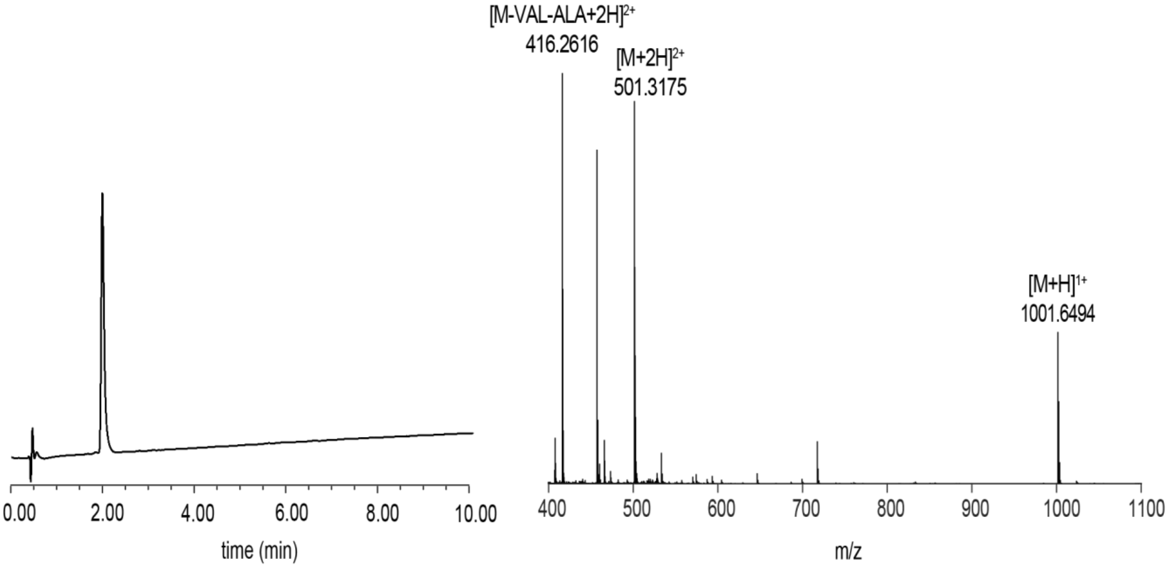

Figure S4. (I) RP-HPLC chromatogram (gradient from 10 to 50% acetonitrile in water over 10 min at 2 ml/min, detection at 214 nm) and (II) high resolution mass spectrum for peptide **L2**.

L2-K

(I) **(II)**

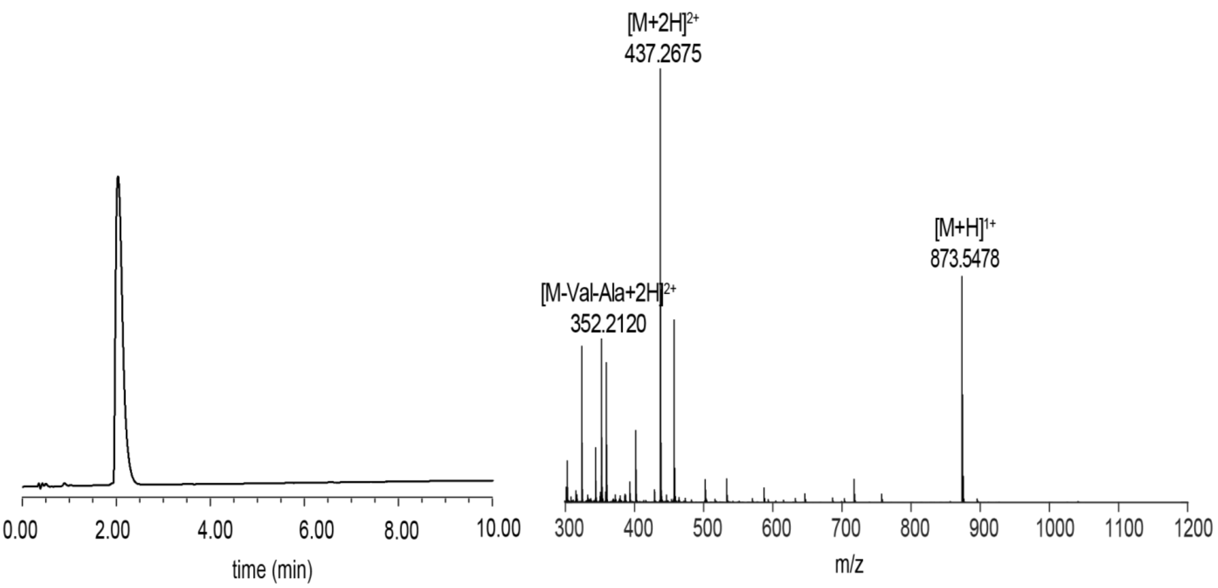

Figure S5. (I) RP-HPLC chromatogram (gradient from 10 to 50% acetonitrile in water over 10 min at 2 ml/min, detection at 214 nm) and (II) high resolution mass spectrum for peptide **L2-K**.

L3

(I) **(II)**

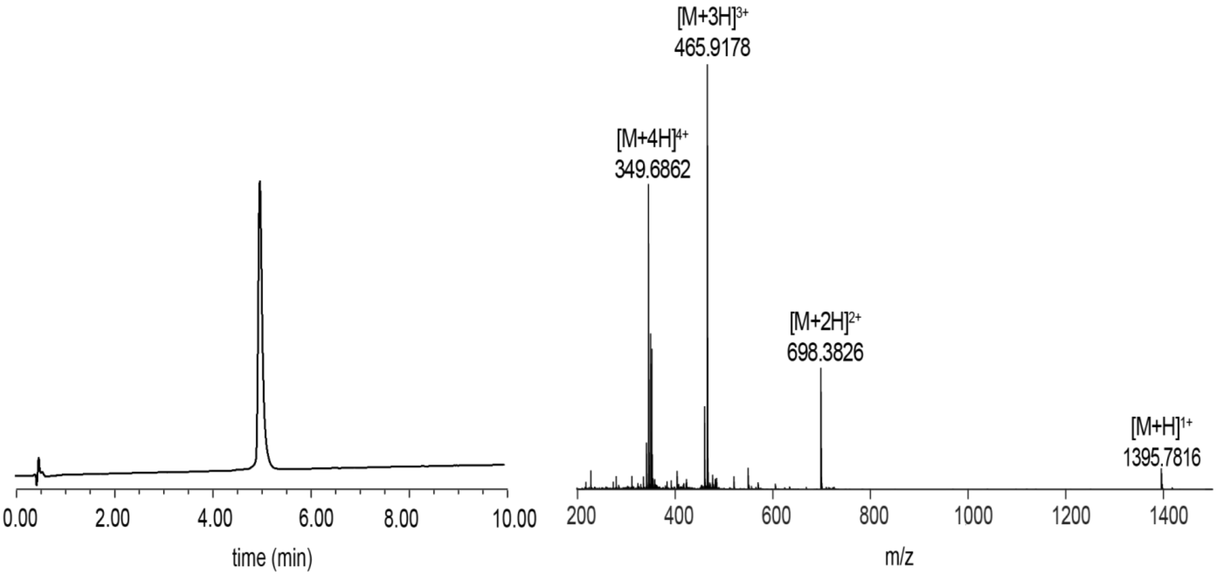

Figure S6. (I) RP-HPLC chromatogram (gradient from 10 to 50% acetonitrile in water over 10 min at 2 ml/min, detection at 214 nm) and (II) high resolution mass spectrum for peptide **L3**.

L3-K

(I) **(II)**

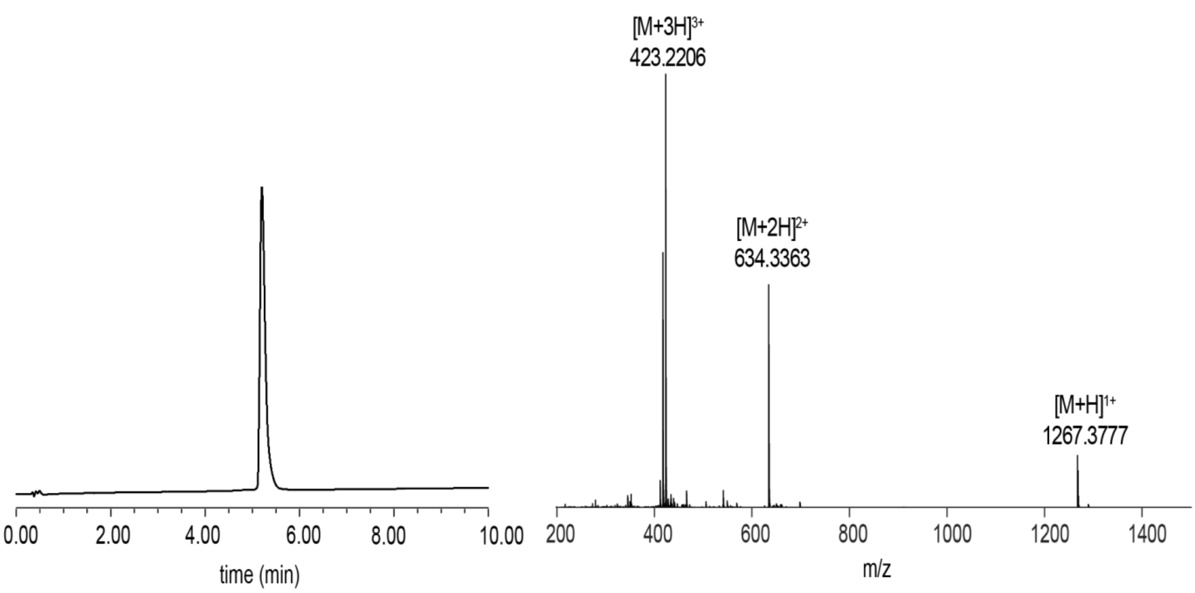

Figure S7. (I) RP-HPLC chromatogram (gradient from 10 to 50% acetonitrile in water over 10 min at 2 ml/min, detection at 214 nm) and (II) high resolution mass spectrum for peptide **L3-K**.

C1

(I) **(II)**

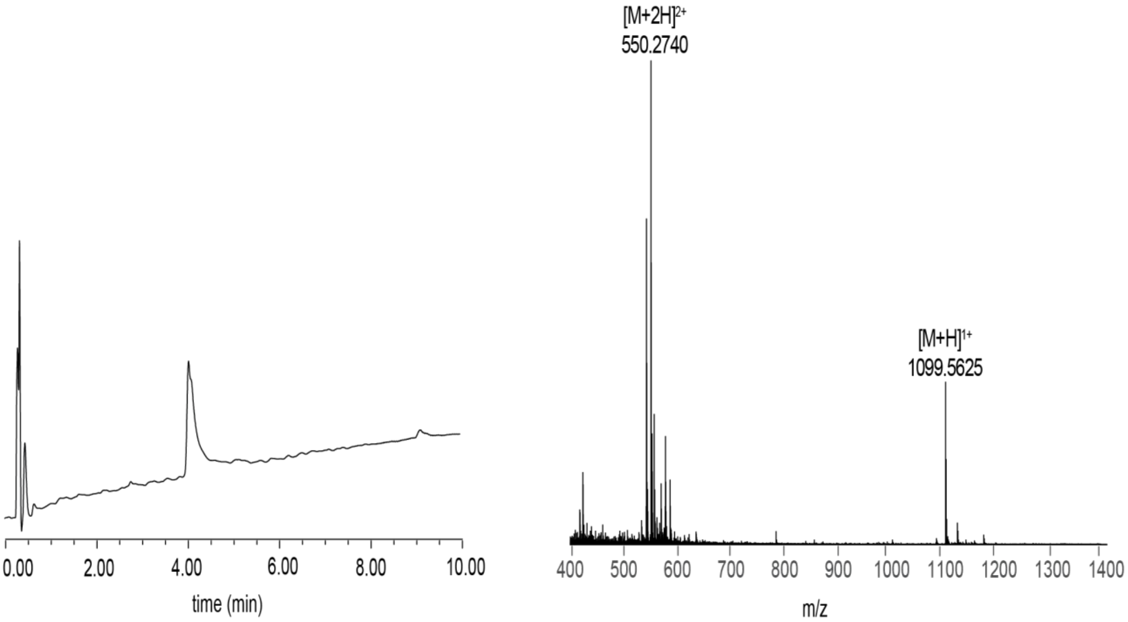

Figure S8. (I) RP-HPLC chromatogram (gradient from 10 to 50% acetonitrile in water over 10 min at 2 ml/min, detection at 214 nm) and (II) high resolution mass spectrum for peptide **C1**.

C2

(I) **(II)**

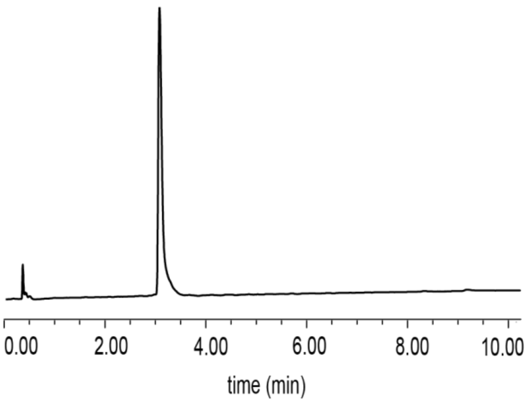

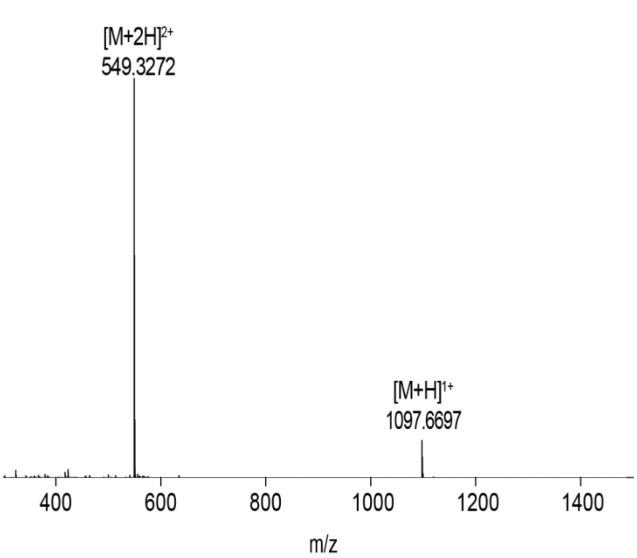

Figure S9. (I) RP-HPLC chromatogram (gradient from 10 to 50% acetonitrile in water over 10 min at 2 ml/min, detection at 214 nm) and (II) high resolution mass spectrum for peptide **C2**.

C3

(I) **(II)**

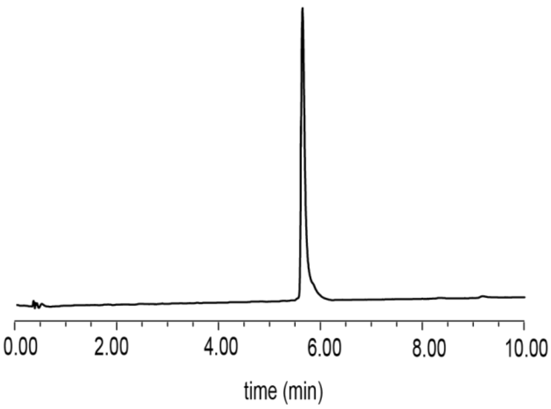

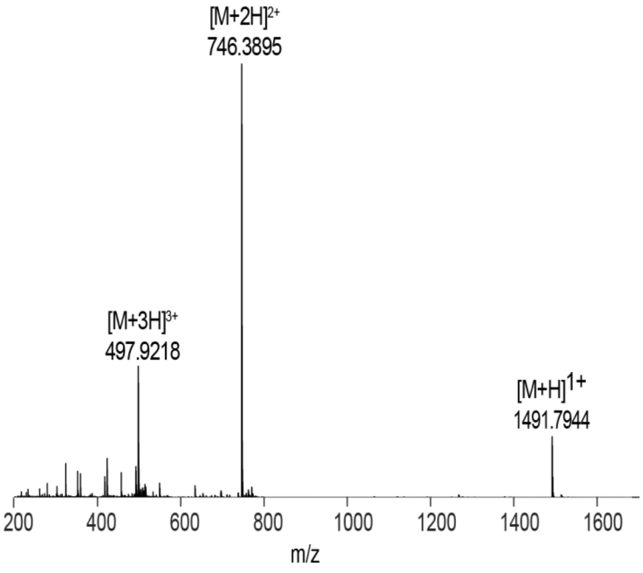

Figure S10. (I) RP-HPLC chromatogram (gradient from 10 to 50% acetonitrile in water over 10 min at 2 ml/min, detection at 214 nm) and (II) high resolution mass spectrum for peptide **C3**.

Amino acid analysis

The method for the quantification of individual amino acids within the synthesized linear peptides (**L1**, **L2** and **L3**) is based on a procedure optimized from previous publication (Baumruck et all 2021).[79] Quantification was done by complete peptide hydrolysis, subsequent ACQ derivation followed by HPLC separation and UV quantification of the individual amino acid signals. External calibration for the quantification was done by measuring peak areas of dilutions of a known amino acid standard. All HPLC injections were done in replicates in order to demonstrate reproducibility and the mean values of both measurements were processed for calculations.

HPLC analysis was performed using MultoHigh 100 RP18 3µ column (125 x 4 mm) at a column temperature of 45°C. Eluent A was sodium acetate buffer (50 mM, pH 5.75) and eluent B was 70% acetonitrile, 30% sodium acetate buffer (50mM, pH 6.0). The gradient method started with an isocratic mixture of 2 % eluent B for 2.5 min followed by a linear gradient of 2 – 30 % eluent B over 70 min and short final linear gradient of 30 – 70 % eluent B for 5 min at a flow rate of 1 mL/min. Chromatograms were extracted and analyzed at 254 nm.

HPLC for linear calibration

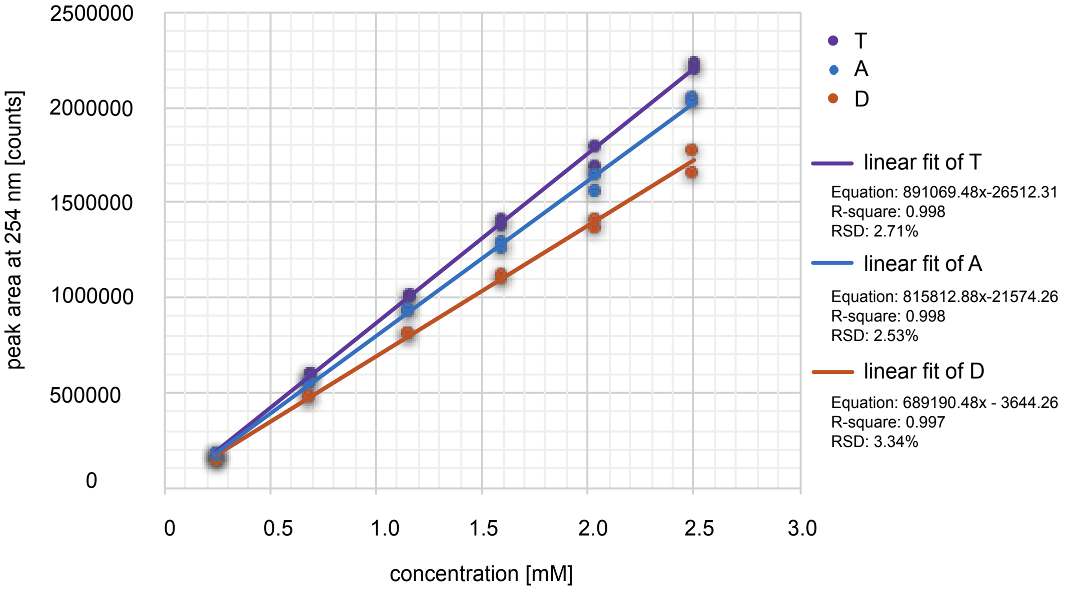

Figure S11. Calibration curves of the amino acids T, A and D used for the peptide content calculation.

Calculation of peptide content

Table S3. Amino acid analysis: HPLC peak areas used for the calculation of the peptide content.

Amino acid	L1		L2		L3	
	Peak area at 254 nm (counts)	Peptide content (%)	Peak area at 254 nm (counts)	Peptide content (%)	Peak area at 254 nm (counts)	Peptide content (%)
Asp	1581960	88				
	1450250					

Thr	1056196	97	1051660	97	1048933	97
	1047519		1052890		1046399	
Ala	1991118	98	2007900	99	1992944	99
	1973285		2011148		2002186	
	**Average**	**94**		**98**		**98**
	**SD**	**5**		**3**		**3**

With the slope, intercept and the average of two measured peak areas at 254 nm the average amino acid concentration (avgCx) for each specific amino acid was calculated. The following equation leads to the amino acid content:

$$\boldsymbol{amino\ acid\ content}\ (\%) = \frac{\boldsymbol{avgCx * 2 * 100}}{\boldsymbol{2.5\ (theore.conc.) * no\ of\ aa\ in\ sequence}}$$

Please note, that ehe average amino acid concentration (avgCx) is multiplied by a factor of 2, because 10 µl of the hydrolyzed and derivatized peptide samples were injected instead of 20 µl as used in the amino acid calibrations.

Circular dichroism spectroscopy

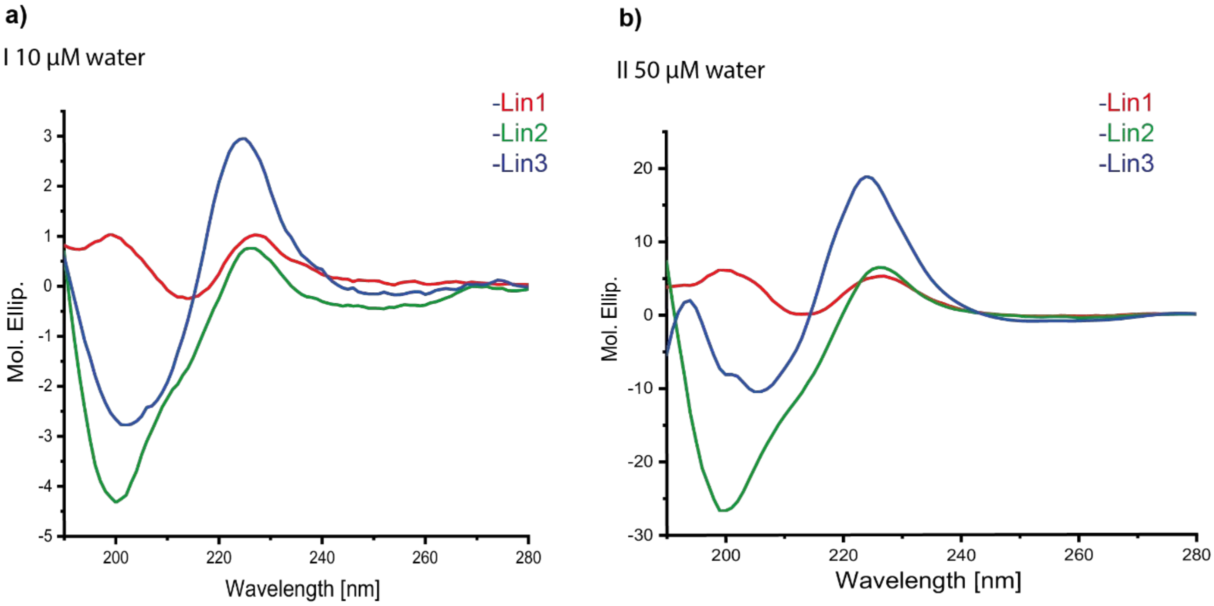

Figure S12. CD spectra of linear synthesized peptides **Lin1** in red, **Lin2** in green and **Lin3** in blue in two different solvents and concentration. **A)** 10µM in water, 293 K, **b)** 50µM in water, 293 K.

NMR

Table S4. Temperature dependence of NH chemical shifts of in H20/D2O 9:1 at 900 MHz.

**Peptide/Residue/NH**	**280(1) K** δ_H**[ppm]**	**290(2) K** δ_H**[ppm]**	**300(3) K** δ_H**[ppm]**	**310(4) K** δ_H**[ppm]**	$\Delta\delta/\Delta T$ **[ppb/K]**
L3/1 Trp/NH	x	x	x	x	x
C3/1 Trp/NH	8.34	8.25	8.20	8.13	-6.80
L1/1 Ala/NH	x	x	x	x	x

L2/1 Ala/NH	8.50	8.42	8.34	8.24	-6.90
L3/2 Ala/NH	8.34	8.27	8.20	8.10	-7.90
C2/1 Ala/NH	8.36	8.32	8.28	8.22	-6.06
C3/2 Ala/NH	8.18	8.11	8.04	7.97	-7.00
L1/2 Val/ NH	8.55	8.49	8.42	8.34	-7.0
L2/2 Val/ NH	x	x	x	x	x
L3/3 Val/ NH	8.22	8.14	8.10	7.96	-8.20
C2/2 Val/ NH	8.15	8.07	8.01	7.94	-9.08
C3/3 Val/ NH	7.78	7.73	7.68	7.55	-7.4
L1/3 Ala/ NH	8.55	8.46	8.38	8.28	-8.90
L2/3 Ala/ NH	8.53	8.47	8.41	8.32	-6.90
L3/4 Ala/ NH	8.37	8.29	8.20	8.12	-8.40
C2/3 Ala/ NH	8.15	8.07	8.01	7.94	-9.08
C3/4 Ala/ NH	8.20	8.12	8.10	7.97	-7.10
L1/4Trp/ NH	8.41	8.31	8.22	8.12	-9.60
L2/4Trp/ NH	8.26	8.17	8.08	7.99	-9.00
L3/5Trp/ NH	8.17	8.09	8.00	7.91	-8.7
C2/4Trp/ NH	8.33	8.22	8.18	8.13	-8.44
C3/5Trp/ NH	8.11	8.04	7.98	7.90	-6.90
L1/5Asp/ NH	8.13	8.09	8.06	8.01	-3.90
L2/5Orn/ NH	8.39	8.33	8.38	8.21	-5.90
L3/6Orn/ NH	8.39	8.34	8.28	8.20	-6.30
C2/5Orn/ NH	8.40	8.34	8.29	8.24	-6.97
C3/6Orn/ NH	8.27	8.20	8.10	8.01	-8.80
L1/6Thr/ NH	8.11	8.08	8.03	7.99	-4.10
L2/6Thr/ NH	8.22	8.15	8.07	7.99	-7.70
L3/7Thr/ NH	8.10	8.02	7.95	7.89	-7.0
C2/6Thr/ NH	8.03	7.99	7.96	7.92	-4.74
C3/7Thr/ NH	8.0	7.94	7.87	7.81	-6.4
L1/7Asp/ NH	8.55	8.50	8.45	8.38	-5.60
L2/7Orn/ NH	8.56	8.49	8.42	8.32	-8.00
L3/8Orn/ NH	8.50	8.42	8.33	8.26	-8.10
C2/7Orn/ NH	8.46	8.40	8.36	8.30	-6.85
C3/8Orn/ NH	8.32	8.25	8.17	8.10	-7.40
L1/8Val/ NH	8.07	8.01	7.95	7.89	-6.00
L2/8Val/ NH	8.37	8.30	8.22	8.12	-8.30
L3/9Val/ NH	8.37	8.25	8.20	8.10	-8.60
C2/8Val/ NH	8.35	8.26	8.30	8.14	-7.87
C3/9Val/ NH	8.27	8.28	8.17	8.07	-6.40
L1/9Lys/ NH	8.41	8.33	8.25	8.17	-8.00
L2/9Lys/ NH	8.84	8.74	8.64	8.53	-10.30
L3/11Lys/ NH	8.28	8.22	8.16	8.05	-7.50
C2/9Lys/ NH	8.58	8.50	8.43	8.36	-9.60
C3/11Lys/ NH	8.38	8.31	8.24	8.17	-7.00
L3/10NTY/ NH	8.45	8.40	8.33	8.26	-6.40
C3/10NTY/ NH	8.62	8.54	8.45	8.36	-8.70

(1) For C2 NH chemical shifts at 275K
(2) For C2 NH chemical shifts at 283K
(3) For C2 NH chemical shifts at 290K
(4) For C2 NH chemical shifts at 298K

Table S5. Energy and structural ensembles of synthesized peptides.

Variables	Peptide				
	**L1**	**L2**	**L3**	**C2**	**C3**
Distance restrains, n#	17	12	35	22	42
Dihedral angle restrains, n	1	1	2	4	5
Structure statistics, kj/mol					
Violations					
Total restrain violation energy	0.75	0.22	2.51	3.23	4.02
Distance restrains	0.51	0.17	2.51	3.15	3.96
Dihedral angle restrains	0.24	0.05	0	0.08	0.06
Total Force field energy (YASARA)	-4827.15	-4030.31	-4190.09	-2776.76	-3150.28
Internal solute energy	-1057.41	-501.001	595.13	-524.13	-363.64
Electrostatic solv. Energy	-3887.06	-3620.88	-4968.42	-2163.76	-2646.75
Van der Waals solv. Energy	117.32	91.57	183.20	-88.88	-139.89
Mean RMSD* (Å), mean±SD					
Backbone global	1.22 ± 0.23	1.46 ±0.41	0.84 ±0.28	0.57 ± 0.22	1.87 ±0.45
Heavy atoms	1.87 ± 0.33	1.77 ± 0.50	0.98 ± 0.34	0.63 ±0.22	2.88 ±0.62

MMC_{99} activity including salt stability measurements

Table S6. Antimicrobial activity of L2 and L3 in the presence of NaCl.[1]

Peptide sin NaCl (mM)	MMC_{99} (µg/ml)								
	S. aureus			*E.coli*			*C. albicans*		
	2h	6h	24h	2h	6h	24h	2h	6h	24h
2L									
0							50	25	25
85	–	–	–	–	–	–	>200	>200	>200
150	–	–	–	–	–	–	>200	>200	>200
3L									
0	12.5	12.5	25	12,5	6.3	6.3	12.5	6.3	12.5
85	>200	>200	(-)[2]	>200	>200	>200	200	100	50
150	>400	>400	(-)[2]	>400	>400	**>**400	>200	>200	>200

[1] Antimicrobial activity was measured as minimum microbicidal concentration (µg/mL) killing ≥99% of the inoculum (MMC_{99}) after 2, 6, and 24 h of incubation in BHI diluted 1/100 (BHI_{100}), containing 85- or 150 mM NaCl. **L2** and **L3** were serially diluted in twofold steps with a starting dilution of 200 µg/ml except for **L3** in 150 mM NaCl, where 400 µg/mL were used for the bacterial strains. All sample dilutions were run in duplicates (**L2**) or triplicates (**L3**).

[2] The growth of *S. aureus* at 24 h in BHI_{100} with 85 or 150 mM NaCl was reduced.

Minimum microbicidal concentration (MMC), killing 99% of the inoculum.

All microorganisms were cultured in brain-heart infusion broth (BHI) overnight on a shaker at 37°C. The cultures were thereafter transferred to fresh BHI broth in volumes constituting 10% of total, and incubated for an additional 2 h to reach exponential growth phase. The bacterial cultures were washed once in BHI diluted 1/100 (BHI_{100}), followed by resuspension in BHI_{100}. The cell suspensions were diluted to a density of $1x10^7$ (bacteria) or $2x10^7$ CFU/ml (yeast), as estimated by optical density measurement at 590 nm.

Peptides were serially diluted by twofold steps in a microtiter plate (Sarstedt, Numbrecht, Germany, 82.1581001) in duplicate or triplicate (200 µl per well), starting with 100 µg/ml as the highest concentration unless otherwise stated. The diluents used were BHI_{100}, and BHI_{100} with 85 or 150 mM NaCl. The bacterial or yeast cell suspensions were added in 10-µl volumes to the wells, giving a final concentration of approximately 5 x 10^5 cells/ml. The initial concentration of the inoculum was checked by viable counts. The microplate was incubated at 37°C in a humid chamber for 24 h. Viability of the inoculum was analysed at 2, 6 and 24 h by culturing 5 µL of the suspension of each well, added as a drop onto blood agar plates (blood agar supplemented with 5% defibrinated horse blood). After incubation overnight at 37 ◦C, the viable count in each drop was recorded, and the concentration of CFU/ml calculated. The MMC of a peptide causing ≥99% reduction of the inoculum was defined as MMC_{99}.

Serum stability

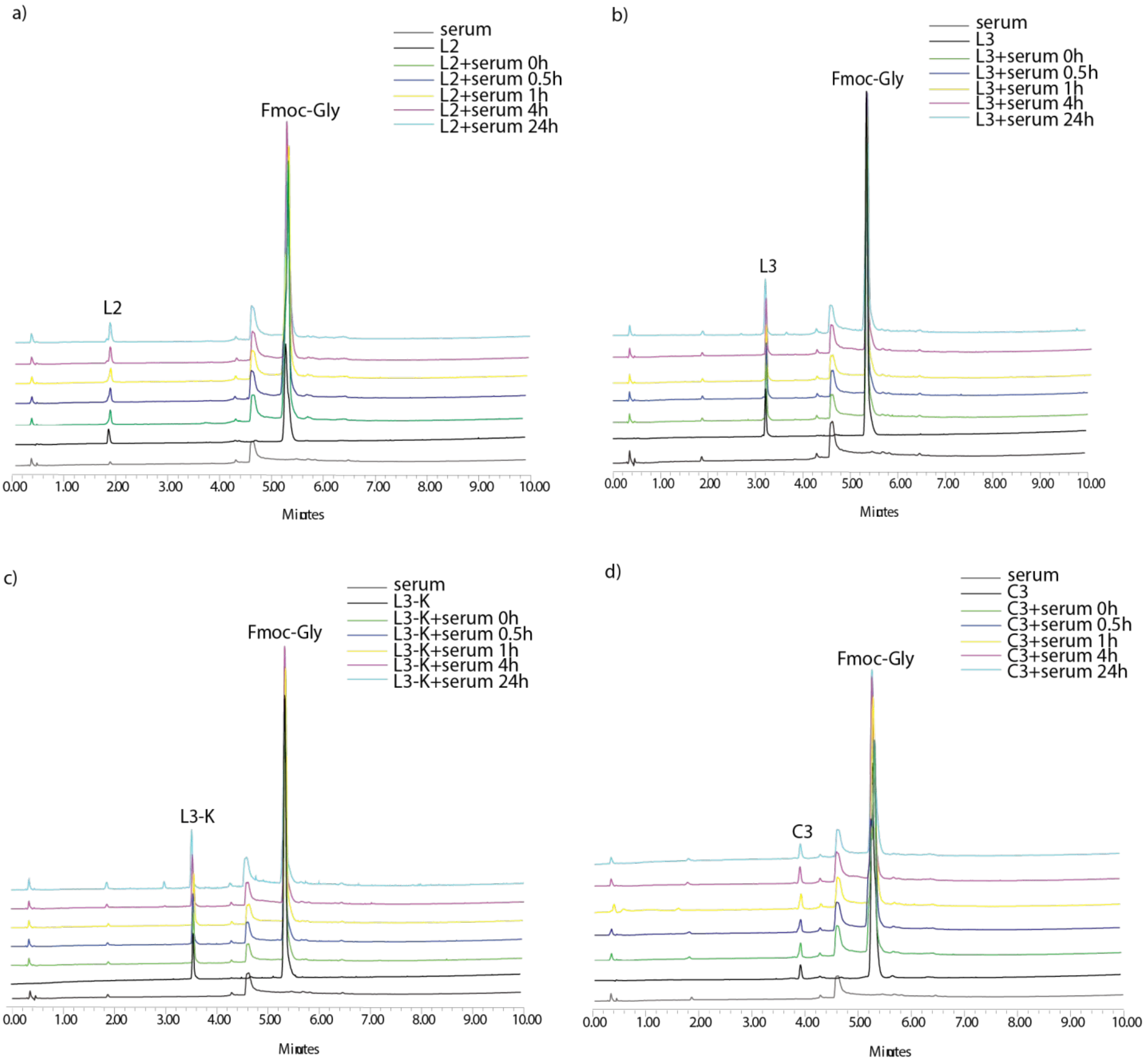

Figure S 133. RP-HPLC chromatogram (gradient from 10 to 90% acetonitrile in water over 10 min at 2 ml/min, detection at 254 nm) of peptides tested for serum stability. A) peptide **L2** B) peptide **L3**. C) peptide **L3-K**. D) peptide **C3**.

Hemolysis

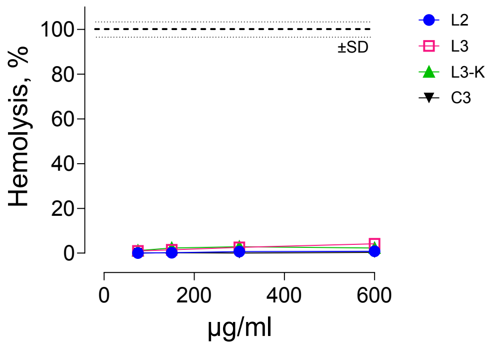

Figure S 144. Haemolytic activity of fresh human erythrocytes from blood donors post peptide exposure. All peptides were analysed with a starting concentration of 600 μg/ml (twofold dilutions). All peptide dilutions were assayed in duplicate. The positive control (100±3 %) consisted of 0.1 % Triton X-100 in PBS.

Bacterial cytological profiling of peptides

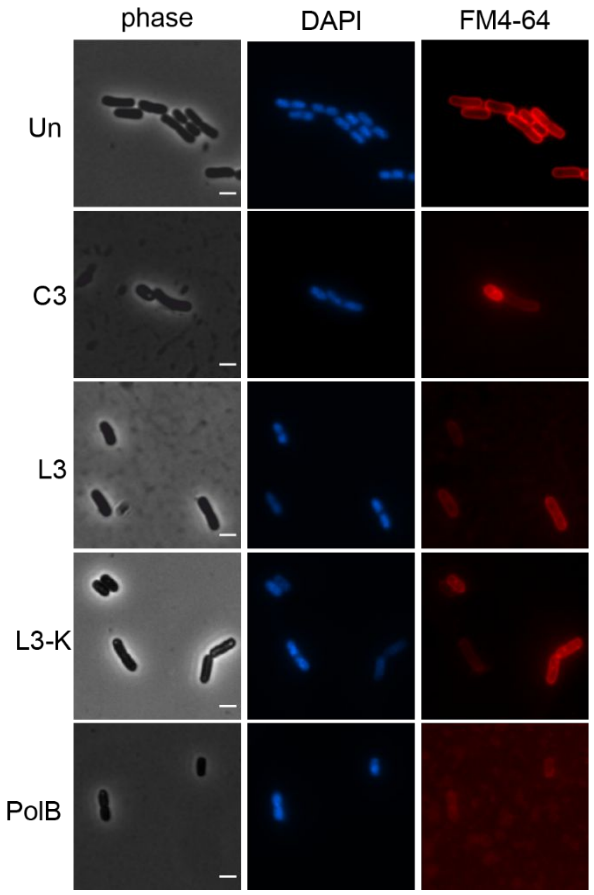

Figure S15. Bacterial cytological profiling of *E. coli* CCUG31246 treated with **C3**, **L3**, **L3-K**, and PolB for 10 min. Scale bar 2 μm.

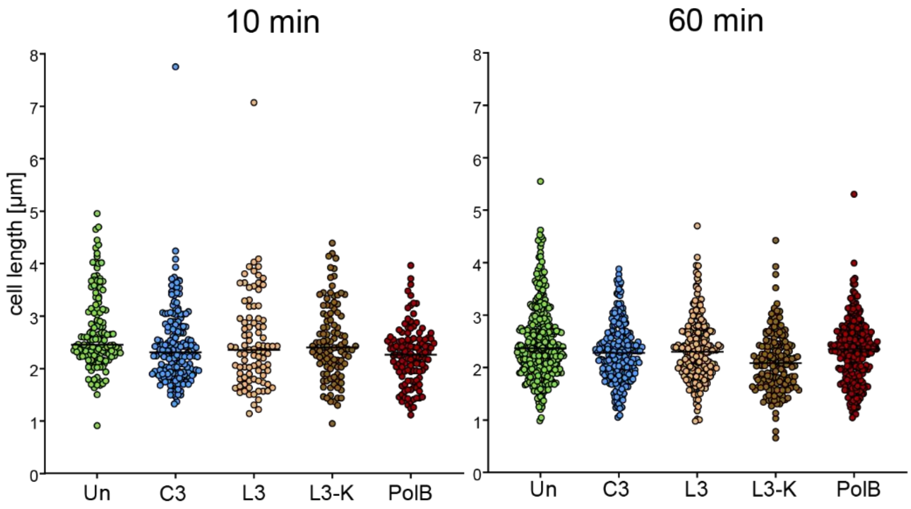

Figure S 16. Cell length measurements of *E. coli* CCUG31246 after 10 and 60 min treatment with **C3 L3, L3-K,** and PolB. Black lines indicate the median of each sample. Cells from three independent biological replicates were pooled for analysis. A minimum of 91 cells was analyzed per sample.
